# Nicotinic acetylcholine receptor (CHRN) expression and function in cultured human adult fungiform (HBO) taste cells

**DOI:** 10.1371/journal.pone.0194089

**Published:** 2018-03-07

**Authors:** Jie Qian, Shobha Mummalaneni, James Larsen, John R. Grider, Andrew I. Spielman, Mehmet Hakan Özdener, Vijay Lyall

**Affiliations:** 1 Department of Physiology and Biophysics, Virginia Commonwealth University, Richmond, VA, United States of America; 2 NYU College of Dentistry, New York, NY, United States of America; 3 Monell Chemical Senses Center, Philadelphia, PA, United States of America; The University of Tokyo, JAPAN

## Abstract

In rodents, CHRNs are involved in bitter taste transduction of nicotine and ethanol. Currently, it is not clear if CHRNs are expressed in human taste cells and if they play a role in transducing the bitter taste of nicotine and ethanol or in the synthesis and release of neurohumoral peptides. Accordingly, we investigated the expression and functional role of CHRNs in HBO cells. Using molecular techniques, we demonstrate that a subset of HBO cells express CHRNs that also co-express TRPM5, T1R3 or T2R38. Exposing HBO cells to nicotine or ethanol acutely or to nicotine chronically induced a differential increase in the expression of CHRN mRNA and protein in a dose- and time-dependent manner. Acutely exposing HBO cells to a mixture containing nicotine plus ethanol induced a smaller increase in CHRN mRNAs relative to nicotine or ethanol treatment alone. A subset of HBO cells responded to nicotine, acetylcholine and ATP with a transient increase in [Ca^2+^]_i_. Nicotine effects on [Ca^2+^]_i_ were mecamylamine sensitive. Brain-derived neurotrophic factor (BDNF) protein was detected in HBO cells using ELISA. Acute nicotine exposure decreased BDNF in HBO cells and increased BDNF release in the medium. CHRNs were also detected in HEK293 cells by RT-PCR. Unlike HBO cells, CHRNs were localized in most of HEK293 cells and majority of HEK293 cells responded to nicotine and ethanol stimulation with a transient increase in [Ca^2+^]_i_. BDNF levels in HEK293 cells were significantly higher than in HBO cells but the nicotine induced release of BDNF in the media was a fraction of the BDNF cellular content. We conclude that CHRNs are expressed in TRPM5 positive HBO cells. CHRN mRNA expression is modulated by exposure to nicotine and ethanol in a dose- and time-dependent manner. Nicotine induces the synthesis and release of BDNF in HBO cells.

## Introduction

In taste buds, a dedicated subset of taste receptor cells (TRCs) detect bitter taste stimuli in the oral cavity. This subset of TRCs express G-protein coupled bitter taste receptors (GPCRs) designated as T2Rs. The requisite downstream intracellular signaling components for bitter taste transduction include the enzyme PLCβ2 and a cation channel TRPM5 [1]. Consistent with this, as described in detail previously [2], TRPM5 knockout (KO) mice lack behavioral and neural responses to quinine, a prototypical bitter taste stimulus. However, TRPM5 KO mice respond to nicotine (Nic), a bitter stimulus, as aversive when compared to water or to quinine. Stimulating the anterior tongue with Nic (1–20 mM) evoked chorda tympani (CT) taste nerve responses in TRPM5 KO mice that were about 40% smaller than those observed in wildtype (WT) mice [2]. Based on these observations, it was proposed that the bitter taste of Nic is sensed by two bitter taste transduction mechanisms. One mechanism comprises the T2R-PLCβ2-TRPM5 pathway that is shared by many bitter stimuli. The second pathway is TRPM5-independent. The presence of a TRPM5-independent pathway for Nic is further supported by the observations that Nic at high concentrations inhibits TRPM5 cation channels overexpressed in HEK cells [3].

As described in detail previously [2], in both WT and TRPM5 KO mice, mecamylamine (Mec), a non-specific blocker of CHRNs, inhibited the CT response to Nic but not to quinine. In behavioral studies, Mec also decreased the aversiveness of Nic in both WT and TRPM5 KO mice. These studies provided the first evidence that TRPM5-independent neural and behavioral responses to Nic in WT and TRPM5 KO mice are partially dependent upon CHRNs. As described in detail previously [4], in addition to Nic, CHRN blockers Mec, dihydro-β-erythroidine (DHβE), and CP-601932 (a partial agonist of α3β4* CHRN) also blocked CT responses to acetylcholine (ACh) and ethanol (ETOH). These results indicate that a component of the bitter taste of Nic, ACh and ETOH is dependent upon the expression of CHRNs in a subset of taste bud cells.

We previously detected the expression of mRNAs for α3, α4, β2, and β4 CHRN subunits in rat fungiform (FF) and circumvallate (CV) taste bud cells [2]. We have now confirmed the expression of CHRN subunit mRNAs and proteins using *in situ* hybridization (ISH), immunocytochemistry (ICC) and qRT-PCR techniques in a subset of rat and mouse CV and FF TRCs. As described in detail previously [5], ISH technique revealed the expression of mRNAs for α7, β2 and β4 CHRN subunits in rat and mouse FF and CV taste bud cells. Specific binding of α3, α4, α7, β2, and β4 antibodies to a subset of WT mouse CV and FF TRCs was observed. In a TRPM5-GFP transgenic mouse model, α3, α4, α7, and β4 antibody binding was localized in a subset of TRPM5 positive TRCs.

As described in detail previously [5], Nic exposure differentially increased the expression of α3, α4, α5, α6, β2 and β4 mRNAs in CV taste bud cells to varying extent. In contrast, ETOH exposure induced an increase in the expression of α5 and β4 mRNAs in CV taste bud cells with a significant decrease in the expression of α3, α6 and β2 mRNAs. These results suggest that changes in CHRN expression may be related to the adaptation of the bitter taste of Nic and ETOH during chronic oral exposure.

Although CHRN subunit expression has been verified in rat and mouse TRCs using several techniques [4, 5], a detailed investigation of CHRN subunit expression and function in human TRCs is lacking. In this regard, in a stably proliferating human taste cell line (HTC-8), RT-PCR demonstrated the gene expression of CHRNA5. In contrast, CHRNA3, CHRNA4, CHRNA6, CHRNB2, and CHRNB4 mRNAs were not detected in HTC-8 cells by RT-PCR [6]. However, at present, it is not clear if beside CHRNA5, HTC-8 cells do not express other CHRNs or their expression levels are too low to be detected. Also it is not clear if CHRN expression in HTC-8 cells can be upregulated following Nic or ETOH exposure. Here, we undertook a detailed investigation of the expression and functional role of CHRNs in HBO cells [7, 8]. RT-PCR studies demonstrated the expression of β-actin, α-gustducin, PLCβ2, T1R3, T2R5, and TRPM5 in HBO cells. Approximately, 60% of HBO cells showed labeling with α-gustducin antibody and 20–30% of HBO cells showed labeling with PLCβ2 antibody. HBO cells also responded with an increase in intracellular Ca^2+^ ([Ca^2+^]_i_) to stimulation with denatonium and sucralose [7, 8]. A subset of HBO cells also express α, β, γ, and δ ENaC subunits [9]. This indicates that HBO cells are an excellent model for investigating the expression and function of receptors involved in human taste reception.

In parallel studies, we also performed CHRNA expression and function in HEK293 cells, an epithelial cell line that has been shown to endogenously express α7 and α5 CHRNs. HEK293 cell line has also been used quite extensively to stably overexpress α3β4, α4β2, and α7 CHRNs [10]. These studies were undertaken to show case the differences in CHRN expression and function in HBO cells and HEK293 cells. Our studies demonstrate that both HBO cells and HEK293 cells endogenously express functional CHRNs that respond with a transient increase in [Ca^2+^]_i_ when stimulated with Nic, ACh or ETOH.

## Materials and methods

### HBO and HEK293 cells

HBO cells were cultured as described earlier [7, 8] and were used for experiments between passage 3 and passage 6. HBO cells can be successfully maintained in culture for at least 12 months without loss of viability. During this time, HBO cells stably display all molecular and physiological features characteristic of mature taste cells. This was demonstrated by RT-PCR and confirmed by sequencing and immunostaining with many taste related molecules. In addition, HBO cells exhibited increase in [Ca^2+^]_i_ in response to appropriate concentrations of taste stimuli representing all five taste qualities, indicating the presence of all appropriate signaling pathways [7, 11–13]. HBO cells, therefore, serve as a model taste system to investigate proliferation, differentiation, and physiological function of human taste cells *in vitro*. HEK293 cells were cultured in a medium contained Dulbecco’s modified Eagle’s medium supplemented with 10% fetal bovine serum, 100 units/ml penicillin G, 100 g/ml streptomycin, and 0.7 mg/ml Geneticin (G418) [3].

### RT-PCR

Total RNA from HBO cells or HEK293 cells was purified by using the TRIzol reagent (cat# 15596018, Thermo Fisher Scientific, MA, USA) and reverse transcripted by using RevertAid First Strand cDNA Synthesis Kit (cat# K1622, Thermo Fisher Scientific, MA, USA). RT–PCR for the detection of CHRN subunits and the other taste receptors were carried out by using MyTaq red mix (cat: BIO-25043, Bioline, Luckenwalde, Germany). Briefly, 2 μg total RNA was used for reverse transcription in a total volume of 20 μl per reaction. Reverse transcription were performed at 42°C x 60 min, then 70°C x 5 min and cooled to 4°C. Subsequently, 100 ng total cDNA was used as template, 35 to 40 cycles of PCR amplification were performed (initial denaturation at 95°C for 1 min, denaturation at 95°C for 15 sec, annealing for 15 sec at 53–60°C, and extension for 10 sec at 72°C). RT-PCR products were subjected to electrophoresis on a 1% agarose gel to determine the expression of CHRN subunits and other taste receptors. Human primers used to detect the presence of mRNAs for the CHRN subunits (CHRNA3, CHRNA4, CHRNA5, CHRNA6, CHRNA7, CHRNB2, and CHRNB4) are shown in Table 1. Human primers used for detecting T1R1, T1R3, PLCβ2, and TRPM5 are shown in Table 2. The primers were synthesized by Thermo Fisher Scientific.

**Table 1:** RT-PCR primers for CHRN subunits.

Gene Product	Sequence	NCBI Reference Sequence
CHRNA3 F	GGTGGACGACAAGACCAAAG	NM_000743.4
CHRNA3 R	GGGAAGTAGGTCACGTCGATT	
CHRNA4 F	GGAGGGCGTCCAGTACATTG	NM_000744.6
CHRNA4 R	GAAGATGCGGTCGATGACCA	
CHRNA5 F	AAAGATGGGTTCGTCCTGTGG	NM_000745.3
CHRNA5 R	CAAACAAAACGATGTCTGGTGTC	
CHRNA6 F	TGAGACTCTTCGCGTTCCTG	NM_001199279.1
CHRNA6 R	ATTTCAGCTTTGTCATACGTCCA	
CHRNB2 F	CAATGCTGACGGCATGTACGA	NM_000748.2
CHRNB2 R	CACGAACGGAACTTCATGGTG	
CHRNB4 F	AACCCGTTACAATAACCTGATCC	NM_000750.4
CHRNB4 R	ATTCACGCTGATAAGCTGGGC	

F = forward; R = reverse

Except for CHRNA6, all the primers for other CHRNs were same as described by Hochheimer et al. [6]

**Table 2:** RT-PCR primers for GPCRs and signaling components.

Gene Product	Sequence	NCBI Reference Sequence
PLCβ2 F	CACCCCAGGGGCTATAAGAG	NM_004573.2
PLCβ2 R	GGACAGGGTTGAGCAGAGAC	
T1R1 F	CGGAGTCTTCTCCTGACTTCA	NM_138697.3
T1R1 R	CCGTGGAGTTGTTTATCTCCTC	
T1R3 F	CCGCCTACTGCAACTACACG	NM_152228.2
T1R3 R	CTAGCACCGTAGCTGACCTG	
T2R38 F	TCCCTGGGAAGGCACATGAG	NM_176817.4
T2R38 R	CAGCACAGTGTCCGGGAATC	
TRPM5 F	GTGACCTGGAGGAGGTGATG	NM_014555.3
TRPM5 R	AGCAGGCTCTTGCGTGAC	

F = forward; R = reverse

### Quantitative real-time PCR (qRT-PCR)

qPCR was used to measure RNA transcripts of CHRN subunits, TRPM5 and PLCβ2. Total RNA was purified by using the TRIzol reagent (cat# 15596018, Thermo Fisher Scientific, MA, USA) and reverse transcripted using High-Capacity cDNA Reverse Transcription Kit (cat# 4368814, Thermo Fisher Scientific, MA, USA). Real-time PCR was conducted using carboxyfluorescein (FAM)-labeled probe sets from Invitrogen, (Carlsbad, CA): GAPDH: Hs02758991_g1, CHRNA3: Hs01088199_m1, CHRNA5: Hs00181248_m1, CHRNA6: Hs02563509_s1, CHRNA7: Hs01063372_m1, CHRNB2: Hs01114010_g1, and CHRNB4: Hs00609520_m1. Results were calculated using the 2−ΔΔCt method based on GAPDH amplification, and normalized to the control group.

### Immunocytochemistry (ICC) studies

ICC studies were performed on HBO cells and HEK293 cells. Cells were plated into 8-well chamber slides (1x10^4^ cells/well) and fixed with ice-cold methanol for 10 min at -20°C. After washing with 1xPBS for 5 min and blocking with 3% (v/v) normal donkey serum in Phosphate Buffered Saline (PBS) for 1 h at room temperature, cells were stained with primary antibodies (1:50 dilution) in 3% (v/v) normal donkey serum in PBS at 4°C overnight. After washing, cells were incubated with fluorescent-conjugated secondary antibodies for 1h at room temperature. Nuclei were visualized with 1 μg/ml DAPI. Images were acquired with a 40X or 63X (1.4 numerical aperture) oil immersion objective and Zeiss LSM 700 confocal laser scanning microscope. Images were processed using Zen 2011 Image Processing Program, ImageJ (NIH software), and Photoshop CS2 software (Adobe Systems). Primary antibodies against AChRα3 (sc-5590), AChRα4 (sc-5591), AChRα5 (sc-376979), AChRα7 (sc-1447), AChRβ2 (sc-11372), TRPM5 (sc-27366), T2R38 (sc-67109), and T1R3 (sc-50352) were obtained from Santa Cruz Biotechnology, CA, USA. AChRα6 (Ca# ab 65168) and AChRβ4 (cat# ab129276) antibodies were obtained from Abcam, MA, USA.

### Immunoblotting and immunoprecipitation

HBO cells were washed with ice-cold PBS and lysed in modified radio-immunoprecipitation assay buffer (50 mM Tris-Cl [pH 7.4], 1% Nonidet P-40, 150 mM NaCl, 1 mM EDTA, 1 mM PMSF, 1 μg/ml each of aprotinin and leupeptin, and 1 mM Na_3_VO_4_). For immunoprecipitation, 40 μl Protein A/G-plus agarose (Santa Cruz Biotechnology) was incubated with 2 μg of antibody for 3h at 4°C with gentle rotation, after removing the antibody by washing the agarose beads once with RIPA buffer, beads were incubated with 1 mg whole cell extracts overnight at 4°C with gentle rotation. The beads were washed with RIPA buffer three times to remove non-specific binding. Immune complexes were eluted by boiling in 2×SDS gel-loading buffer for 5 min. For immunoblotting, 20–50 μg protein was resolved by 10% SDS-PAGE and transferred to nitrocellulose membranes. Membranes were immunoblotted with primary antibodies, followed by HRP-conjugated secondary antibodies. Reactions were visualized by enhanced chemiluminescence reagents (Amersham Biosciences, Piscataway, NJ).

### Ca^2+^-imaging

HBO and HEK cells were grown on glass coverslips (Warner Instruments, Hamden, CT, USA) and washed three times with Ringer’s solution containing (in mM): 140 NaCl, 5 KCl, 1 CaCl_2_, 1 MgCl_2_, 10 glucose, 10 HEPES, pH 7.4 The cells were incubated with 16 μM Fura-2-AM (acetoxy methyl ester from Molecular Probes, Eugene, OR, USA) for 90–120 min at room temperature. Cells were washed with Ringer’s solution and coverslips were mounted in an experimental chamber (RC-26GLP, Warner Instruments; 0.7 ml volume) that fitted on to a Series 20 Chamber Platform (Warner Instruments). The cells were visualized through a water immersion 40X objective (Zeiss; 0.9 NA) with a Zeiss Axioskope 2 plus upright fluorescence microscope. The cells were imaged with a set-up consisting of a cooled CCD camera (Imago, Photonics) attached to an image intensifier (VS4-1845, VideoScope), an epifluorescent light source (Polychrome 5, Photonics), dichroic filter (415 nM), and 510 emission filter (40 nM band pass). The cells were alternately excited with 340 nM and 380 nM and emitted light was imaged at 15s intervals. The temporal changes in fluorescence intensity ratio (FIR; F_340_/F_380_) in individual cells was analyzed using imaging software TILL Vision V3.3 (TILL Photonics, Martinsried, Germany). The changes in FIR were monitored under control conditions and after treating the cells with 0.01 to 1.0 mM nicotine or 10–50 mM ETOH in Ringer’s solution in the absence or presence of 50 μM Mec (Sigma-Aldrich). The FIR values in each cell were normalized to 1 under control conditions [7, 8, 14].

### Enzyme-linked immunosorbent assay for BDNF

BDNF was measured in HBO cells and HEK293 cell lysates and culture medium via a sandwich ELISA using the Promega Emax immune assay (Promega Corporation, Madison, WI, USA) according to the manufacturer’s protocol. ELISA plates were coated with anti-BDNF mouse antibody (mAb; 1:1000) and incubated overnight at 4°C. Next day the plates were washed and blocked with Blocking Buffer (Promega). BDNF standard or sample (100 μl) was added to each well and incubated with shaking for 2h at room temperature. The plates were washed and anti-Human BDNF pAb (1:500; 100 μl) was added to each well and incubated with shaking for 2h at room temperature. After washing, 100 μl of diluted anti-IgY HRP (horseradish peroxidase conjugate; 1:200) was added to each well and developed with TMB (3,3’,5,5’-tetramethylbenzidine) solution. The reaction was stopped by adding 100 μl 1N HCl. The absorbance at 450 nm was measured using a VICTOR 2 plate reader and the concentration of BDNF in the samples was calculated from the standard curve and expressed as pg/2*10^6^ cells as described in detail previously [14, 15].

## Results

### Localization of CHRNs using RT-PCR and Western blots

#### Studies using HBO cells

**Fig 1:**
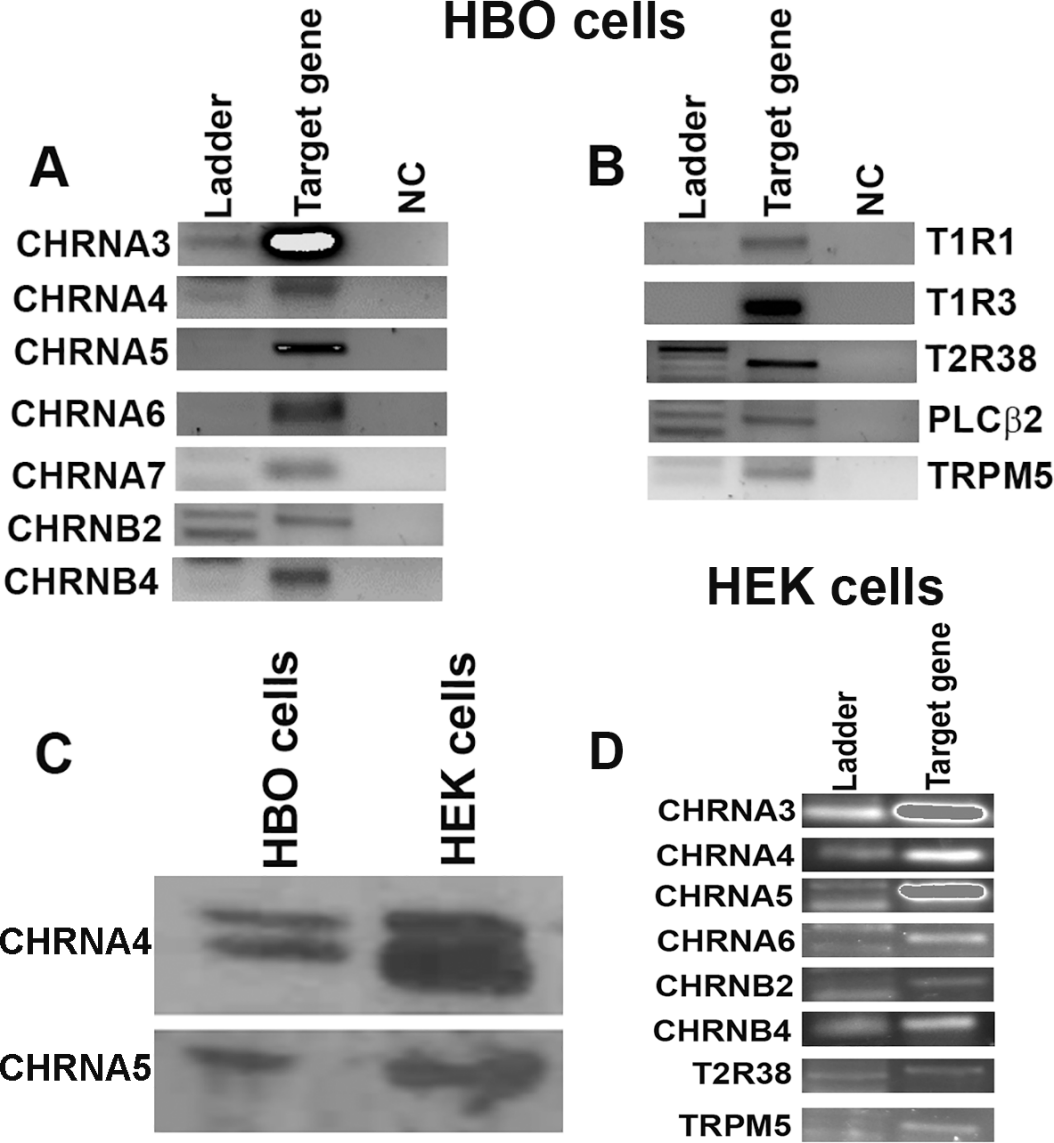
Expression of CHRN subunits, taste receptors, and intracellular signaling intermediates in HBO and HEK293 cells. Consensus primers to amplify CHRN subunits, GPCRs, and downstream signaling intermediate were designed based on the published sequences and are shown in Tables 1 and 2. **(A)** Based on the predicted sizes of the PCR products (Table 1), mRNAs for the CHRNA3, CHRNA4, CHRNA5, CHRNA6, CHRNA7, CHRNB2, and CHRNB4 were detected in HBO cells. **(B)** In addition, mRNAs for T1R1, T1R3, T2R38, PLCβ2, and TRPM5 were detected in HBO cells. **(C)** In Western blot analysis using specific AChRα4 and AChRα5 antibodies, the expression of α4 and α5 proteins were detected in HBO and HEK293 cell lysates. **(D)** Based on the predicted sizes of the PCR products (Table 1), mRNAs for the CHRNA3, CHRNA4, CHRNA5, CHRNA6, CHRNB2 and CHRNB4 were detected in HEK293 cells. In addition, mRNAs for T2R38 and TRPM5 were detected.

From RNA isolated from HBO cells, using RT-PCR, we detected the mRNAs of CHRNA3, CHRNA4, CHRNA5, CHRNA6, CHRNA7, CHRNB2, and CHRNB4 (Fig 1A). In addition, we detected the mRNAs for T1R1 (a component of the umami taste receptor), T1R3 (a component of the sweet taste receptor and the umami taste receptor), T2R38 (a bitter taste receptor), PLCβ2 (an enzyme essential for the transduction of sweet, bitter and umami taste) and TRPM5 (a cation channel essential for the transduction of bitter, sweet and umami taste) (Fig 1B) [7, 8]. Western blot analysis of HBO cell lysate demonstrated the presence of CHRNA4 and CHRNA5 proteins (Fig 1C). We have previously shown the expression of α-, β-, γ-, and δ-ENaC mRNAs and δ-ENaC protein in HBO cells [9]. Taken together, these results indicate that in addition to the classical taste receptors and their downstream signaling effectors, HBO cells express CHRNs.

#### Studies using HEK293 cells

From RNA isolated from HEK293 cells, using RT-PCR technique, we detected the mRNAs for CHRNA3, CHRNA4, CHRNA5, CHRNA6, CHRNB2, and CHRNB4 (Fig 1D). In addition, we detected the mRNAs for T2R38 and TRPM5 (Fig 1D). Western blot analysis of HEK293 cell lysate demonstrated the presence of CHRNA4 (α4) and CHRNA5 (α5) proteins (Fig 1C). HEK293 cells have been shown to endogenously express α7 and α5 CHRNs [10].

### ICC studies

#### Studies using HBO cells

**Fig 2:**
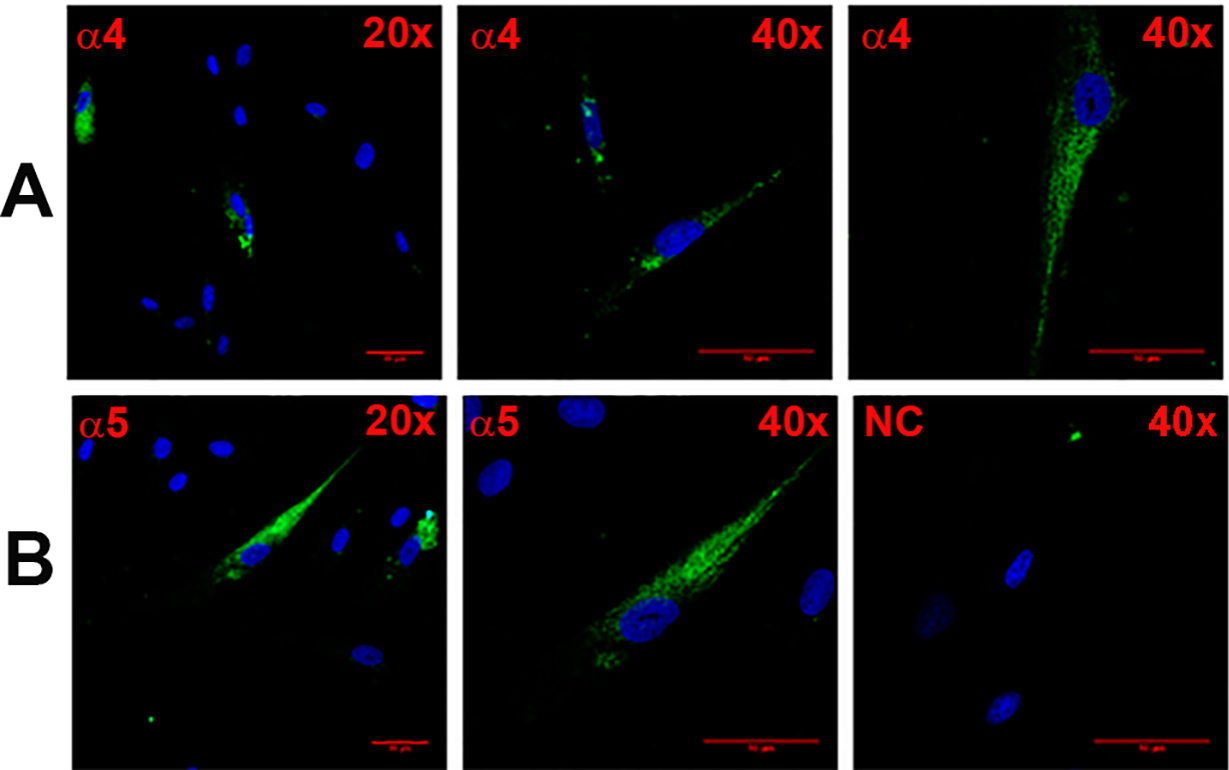
Immunofluorescence staining of CHRNA4 and CHRNA5 in HBO cells. Shows immunostaining of CHRNA4 **(A; α4)** and CHRNA5 **(B; α5)** in HBO cells using 20x and 40x objectives (total 200x and 400x magnification). The panels show merged confocal images of DAPI (blue) and secondary antibody fluorescence (green). The negative control (NC) without primary antibody is also shown. The horizontal red lines represent 10 μm.

**Fig 3:**
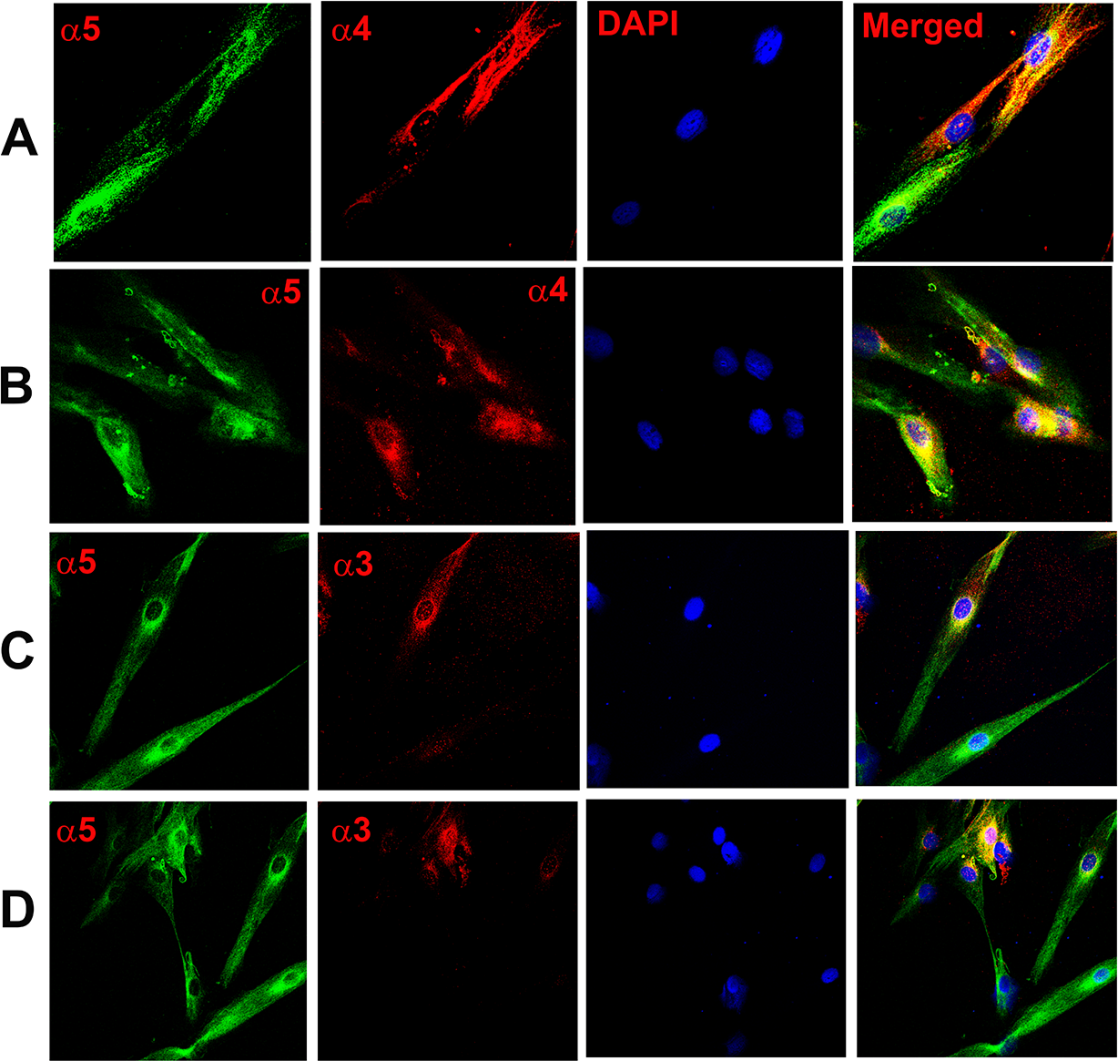
Co-localization of CHRNA subunits in HBO cells. Dual immunostaining was used to co-localize CHRNA subunits in individual HBO cells. **(A and B)** Show immunostaining of CHRNA5 **(α5)** with CHRNA4 **(α4)**. The panels show confocal images of α5 (green), α4 (red), DAPI (blue), and merged images of DAPI and dual fluorescence labels. **(C and D)** Show immunostaining of CHRNA5 **(α5)** with CHRNA3 **(α3)**. The panels show confocal images of α5 (green), α3 (red), DAPI (blue), and merged images of DAPI and dual fluorescence labels.

In a representative slide (Fig 2A; α4), at lower magnification, specific staining of AChRα4 antibody was observed in 3 out of 14 cells in the viewing field. At higher magnification, antibody binding was observed in individual HBO cells. In another representative slide (Fig 2B; α5), at lower magnification, AChRα5 antibody binding was observed in 2 out of 11 cells. At higher magnification, antibody binding was observed in individual HBO cells. In another representative slide (Fig 3C and 3D; α3), AChRα3 antibody demonstrated specific binding to a subset of HBO cells.

**Fig 4:**
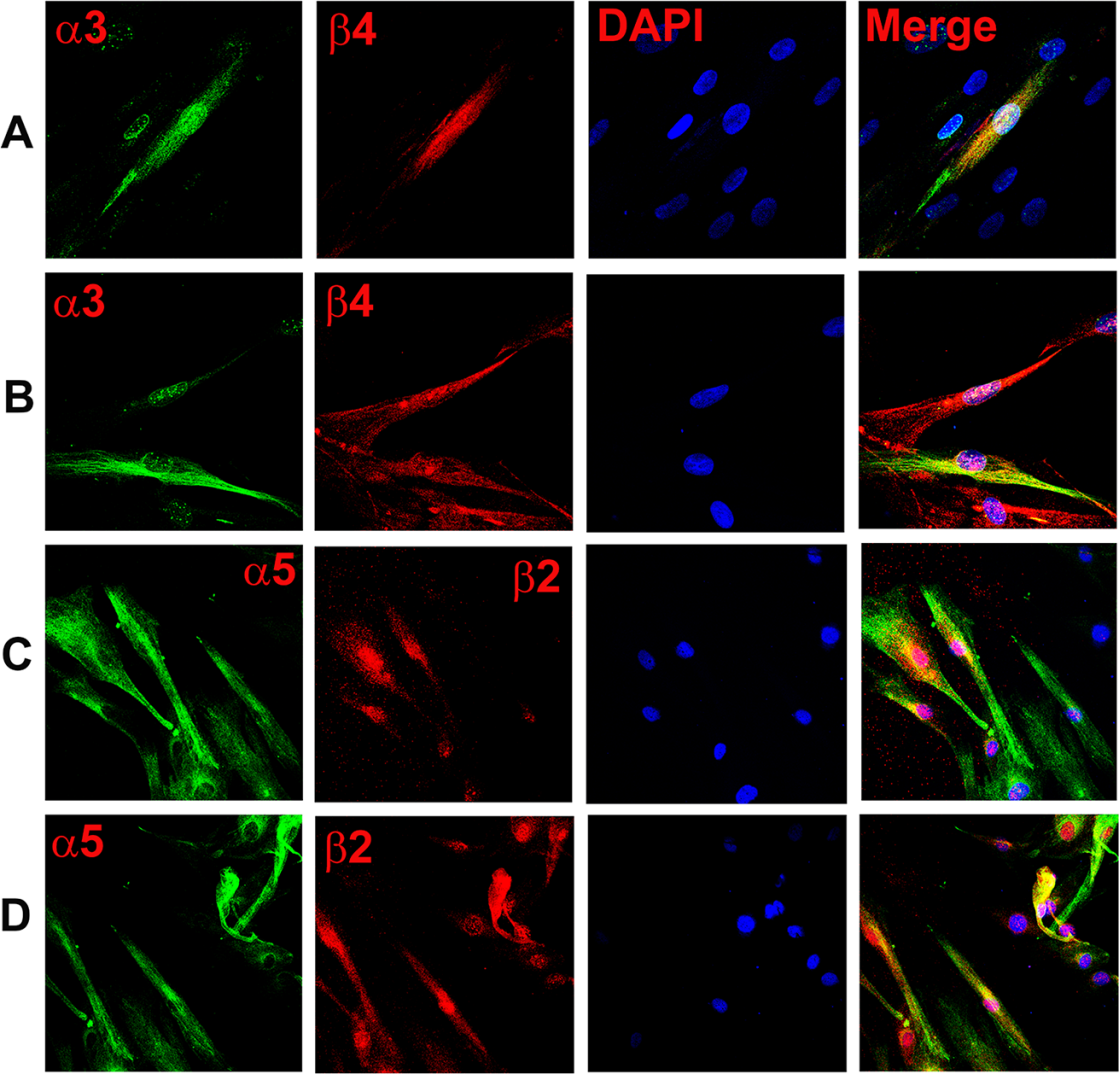
Co-localization of CHRNA and CHRNB subunits in HBO cells. Dual immunostaining was used to co-localize CHRNA and CHRNB subunits in individual HBO cells. **(A and B)** Show immunostaining of CHRNA3 **(α3)** with CHRNB4 **(β4)**. The panels show confocal images of α3 (green), β4 (red), DAPI (blue), and merged images of DAPI and dual fluorescence labels. **(C and D)** Show immunostaining of CHRNA5 **(α5)** with CHRNB2 **(β2)**. The panels show confocal images of α5 (green), β2 (red), DAPI (blue), and merged images of DAPI and dual fluorescence labels.

Dual labelling with AChRα4 and AChRα5 antibodies demonstrated that CHRNA5 (α5) co-localizes with CHRNA4 (α4) in a subset of HBO cells (Fig 3A and 3B; α5, α4). Dual labelling with AChRα5 and AChRα3 antibodies demonstrated that CHRNA5 (α5) co-localizes with CHRNA3 (α3) in a subset of HBO cells (Fig 3C and 3D; α5, α3). Dual labelling with AChRα3 and AChRβ4 antibodies demonstrated that CHRNA3 (α3) co-localizes with CHRNB4 (β4) (Fig 4A and 4B; α3, β4). Dual labelling with AChRα5 and AChRβ2 antibodies demonstrated that in a subset of HBO cells, CHRNA5 (α5) co-localizes with CHRNB2 (β2) (Fig 4C and 4D; α5, β2). These results indicate multiple CHRN subunits are expressed in the same subset of HBO cells.

**Fig 5:**
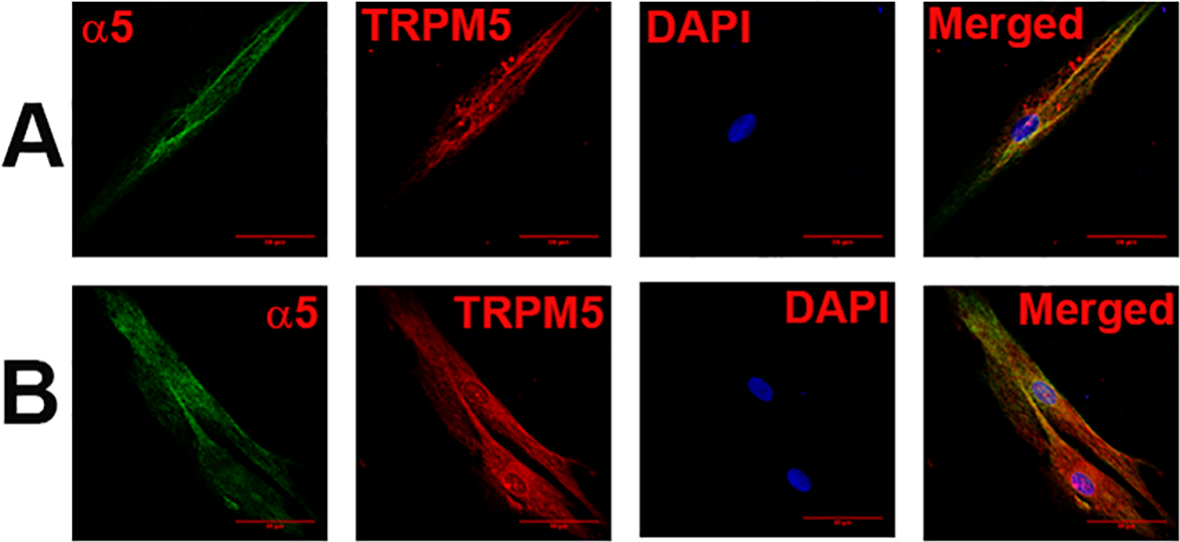
Co-localization of CHRNA5 subunit with TRPM5 in HBO cells. Dual immunostaining was used to co-localize CHRNA5 **(α5)** subunit with **TRPM5** in individual HBO cells. **(A and B)** Show confocal images of α5 (green), TRPM5 (red), DAPI (blue), and merged images of DAPI and dual fluorescence labels.

**Fig 6:**
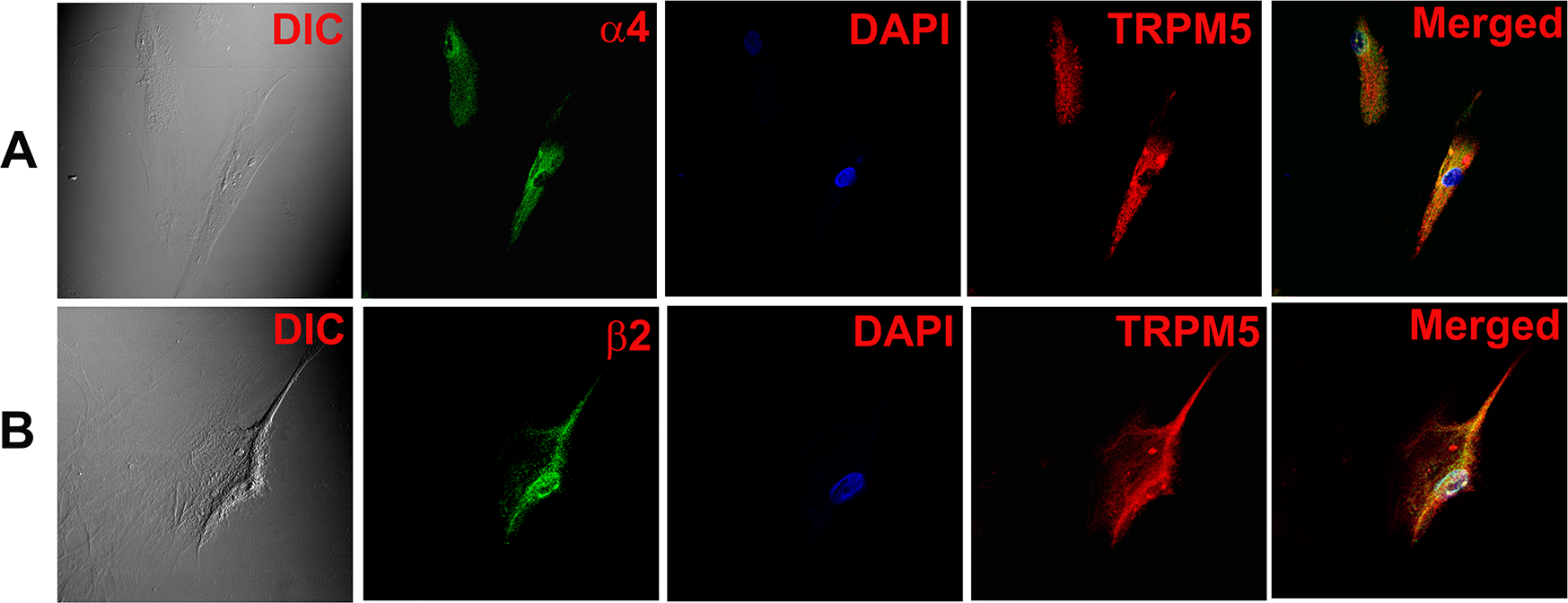
Co-localization of CHRNA4 and CHRNB2 subunits with TRPM5 in individual HBO cells. Dual immunostaining was used to co-localize CHRNA4 **(α4)** or CHRNB2 **(β2)** subunit with **TRPM5** in individual HBO cells. **(A)** Show confocal images of DIC, α4 (green), DAPI (blue), TRPM5 (red), and merged images of DAPI and dual fluorescence labels. **(B)** Show confocal images of DIC, β2 (green), DAPI (blue), TRPM5 (red), and merged images of DAPI and dual fluorescence labels.

**Fig 7:**
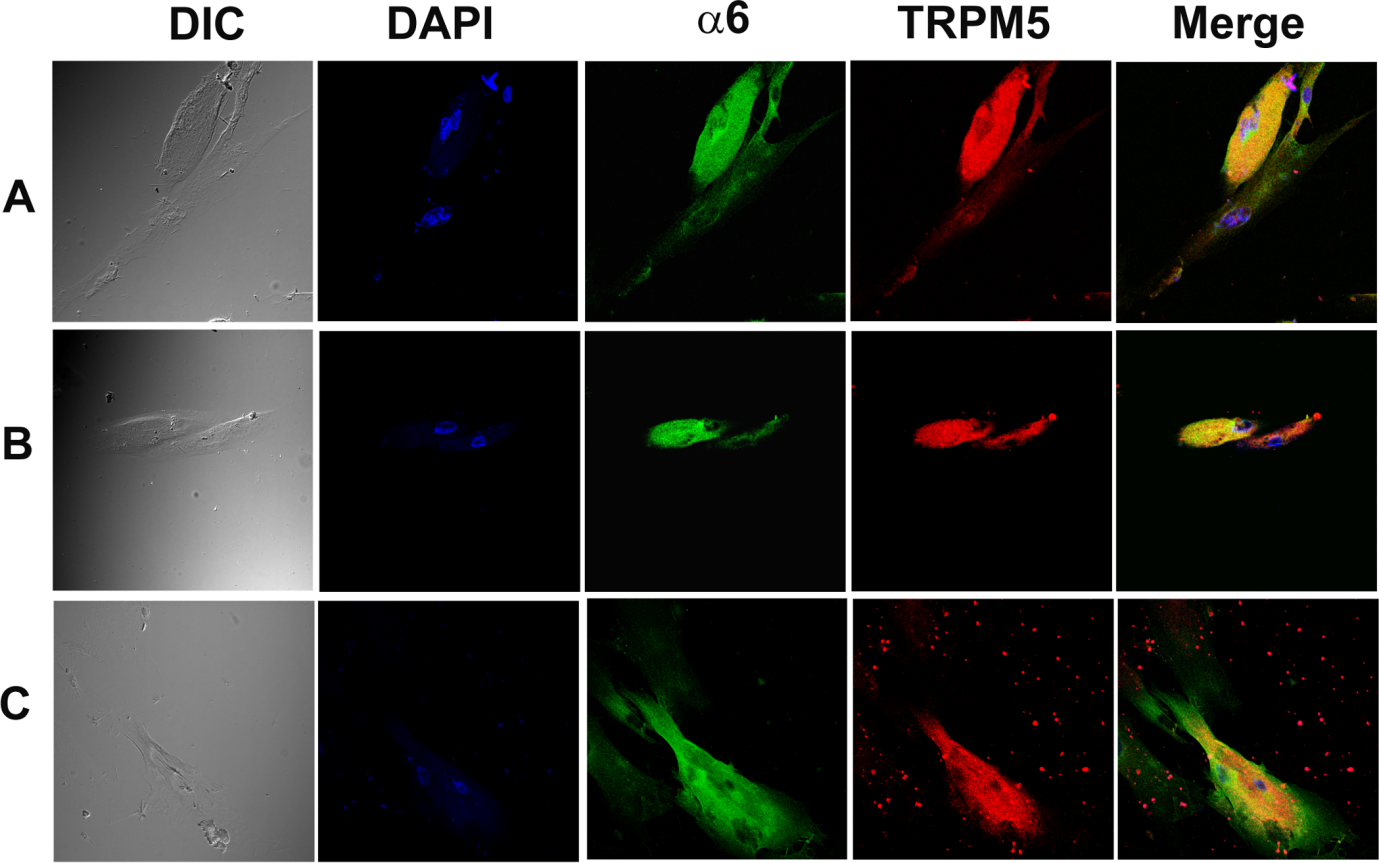
Co-localization of CHRNA6 subunit with TRPM5 in individual HBO cells. Dual immunostaining was used to co-localize CHRNA6 **(α6)** subunit with TRPM5 in individual HBO cells. **(A, B and C)** Show confocal images of DIC, DAPI (blue), α6 (green), TRPM5 (red), and merged images of DAPI and dual fluorescence labels.

In additional HBO cells, dual labelling with AChRα5 and TRPM5 antibodies demonstrated that CHRNA5 (α5) co-localizes in cells that also express TRPM5 (Fig 5A and 5B; α5, TRPM5). Dual labeling with AChRα4 and TRPM5 (Fig 6A) or AChRβ2 and TRPM5 (Fig 6B) antibodies demonstrated that CHRNA4 (α4) and CHRNB2 (β2) subunits co-localize in cells expressing TRPM5. Dual labeling with AChRα6 and TRPM5 antibodies demonstrated that CHRNA6 (α6) subunit co-localize in cells expressing TRPM5 (Fig 7A, 7B and 7C).

**Fig 8:**
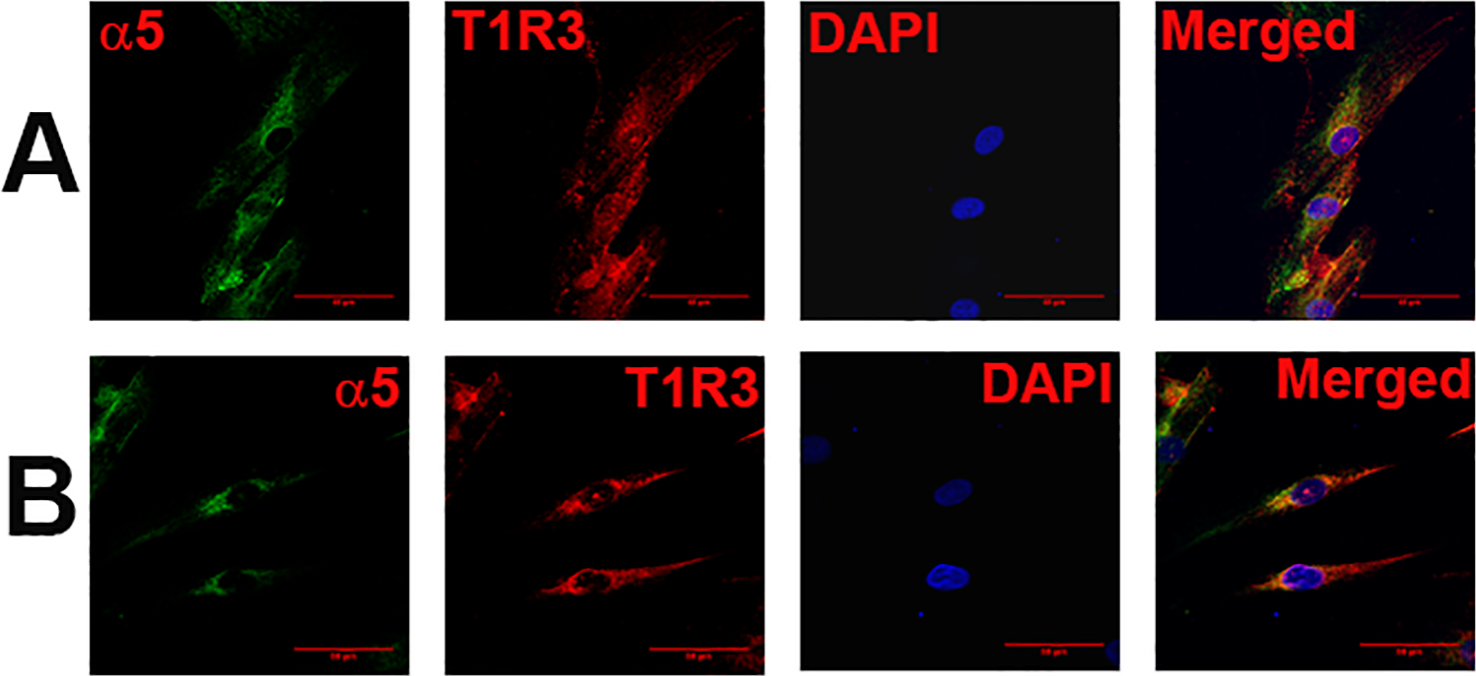
Co-localization of CHRNA5 subunit with T1R3 in HBO cells. Dual immunostaining was used to co-localize CHRNA5 **(α5)** subunit with **T1R3** in individual HBO cells. **(A and B)** Show confocal images of α5 (green), T1R3 (red), DAPI (blue), and merged images of DAPI and dual fluorescence labels.

**Fig 9:**
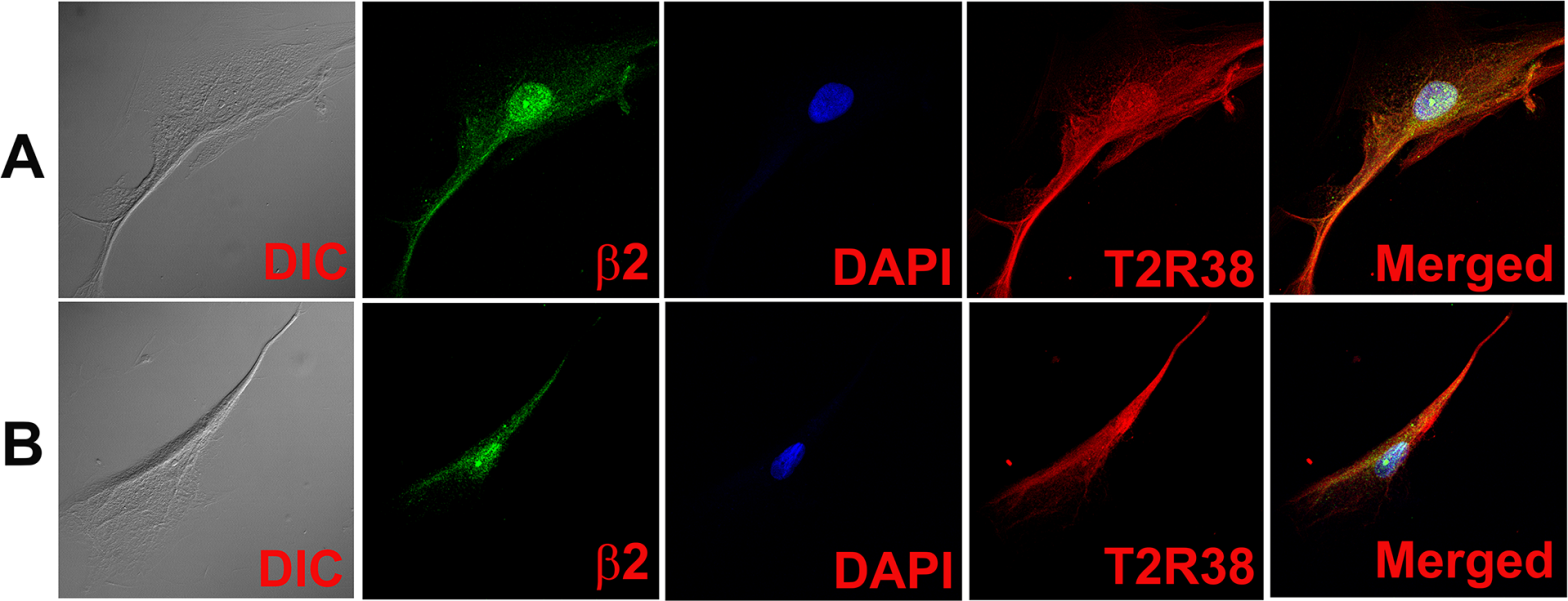
Co-localization of CHRNB2 with T2R38 in HBO cells. Dual immunostaining was used to co-localize CHRNB2 **(β2)** with **T2R38** in individual HBO cells. **(A and B)** Show confocal images of DIC, β2 (green), DAPI (blue), T2R38 (red), and merged images of DAPI and dual fluorescence labels.

Dual labeling with AChRα5 and T1R3 antibodies demonstrated that CHRNA5 (α5) subunit co-localizes in cells expressing T1R3 (Fig 8A and 8B; α5, T1R3). Dual labeling with AChRβ2 and T2R38 antibodies demonstrated that CHRNB2 (β2) subunit co-localizes in cells expressing T2R38 (Fig 9A and 9B; β2, T2R38). Taken together, the above results show that CHRNs are expressed in TRPM5 positive cells that also express T1R3 or T2R38.

We have recently demonstrated that in rat and mouse CV and FF taste bud cells, CHRNs are expressed in a subset of cells that also express TRPM5 [5]. Taken together, the above results indicate that HBO cells faithfully represent the distribution of taste receptors observed in intact taste buds and serve as an excellent model for investigating human taste receptor expression and function.

#### Studies using HEK293 cells

**Fig 10:**
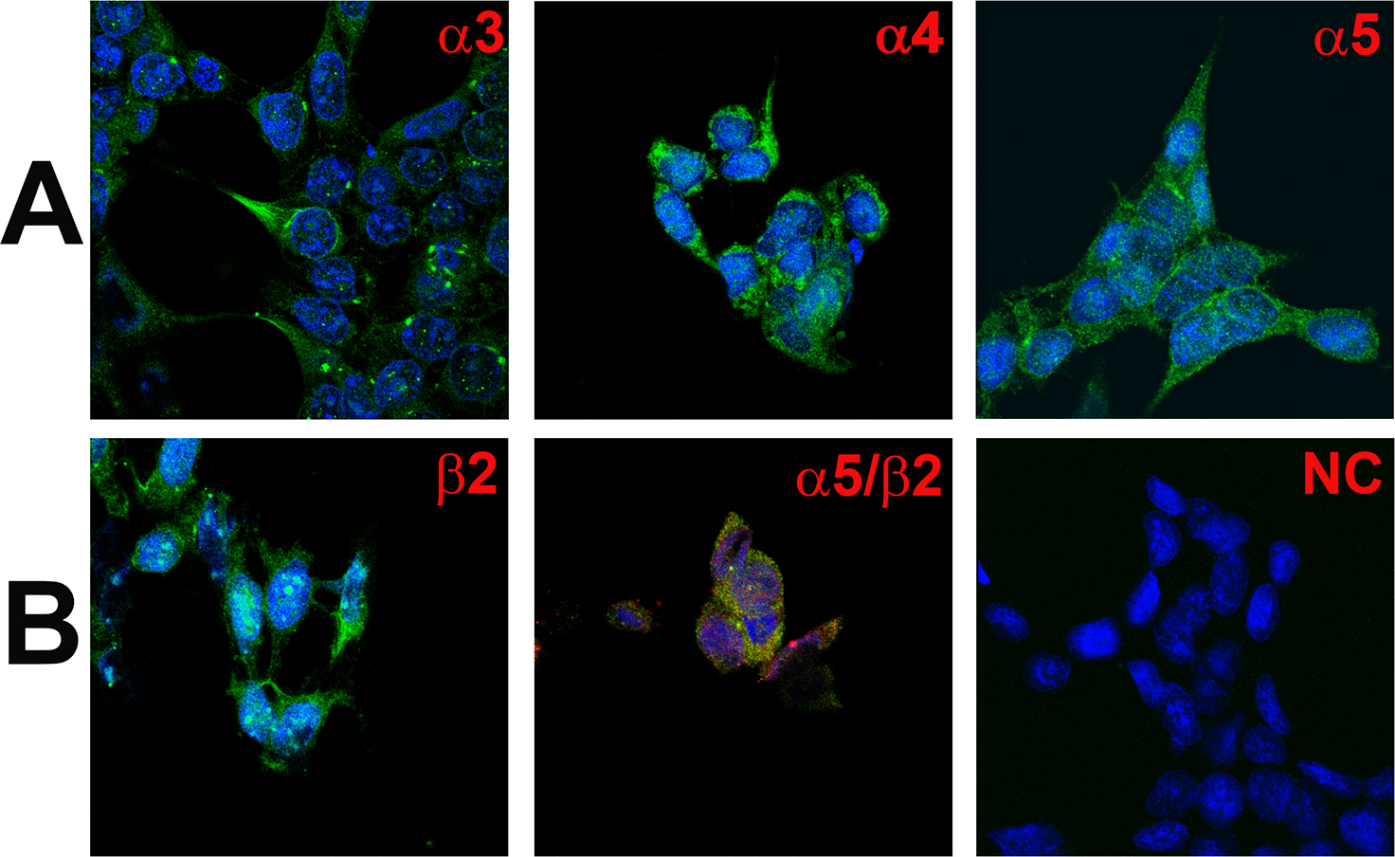
Immunofluorescence staining of CHRNA and CHRNB subunits in HEK293 cells. **(A)** Shows immunostaining of CHRNA3 **(α3)**, CHRNA4 (α4), and CHRNA5 **(α5)** in HEK293 cells. **(B)** Shows immunostaining of CHRNB2 **(β2)** in HEK293 cells. The panels show merged confocal images of DAPI (blue) and secondary antibody fluorescence (green). The negative control **(NC)** without primary antibody is also shown. Dual immunostaining was used to co-localize CHRNA5 **(α5)** with CHRNB2 **(β2)**. **(B, α5/β2)** Shows immunostaining of CHRNA5 (α5; green) with CHRNB2 (β2; red). The panel shows merged confocal images of DAPI and dual fluorescence labels.

Unlike HBO cells, most of the HEK293 cells (Fig 10) demonstrated specific binding to AChRα3 (A; α3), AChRα4 (A; α4), AChRα5 (A; α5), and AChRβ2 (B; β2) antibodies. In addition CHRNA5 was found to co-localize in cells that also express CHRNB2 (Fig 10B; α5/β2). These results indicate that most of the HEK293 express CHRN subunits.

### Localization of CHRN mRNAs in HBO cells using single cell PCR

**Fig 11:**
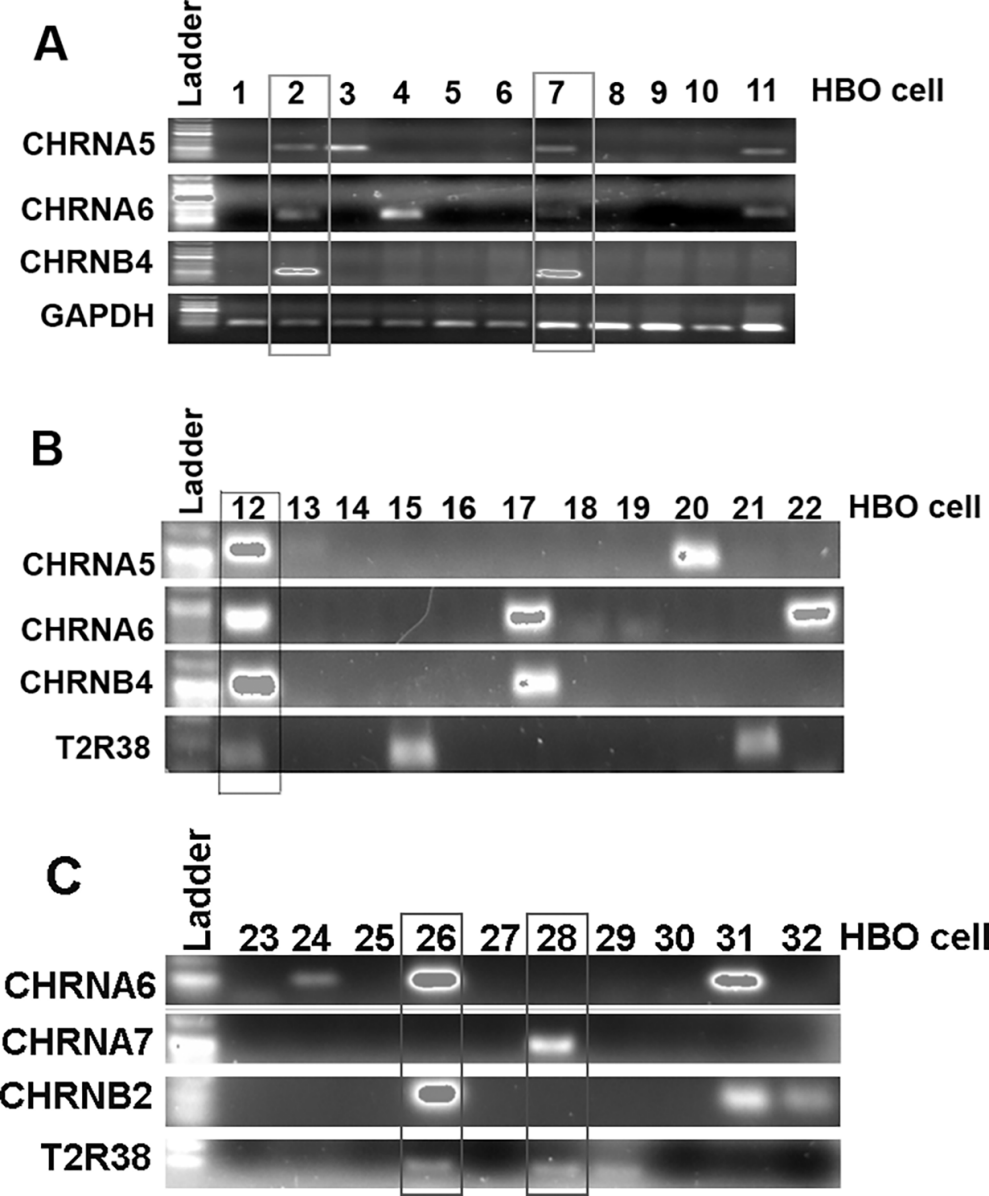
Expression analysis of CHRN subunits using single cell PCR. **(A, B, C)** Single cell real-time RT-PCR technique was used to perform expression analysis of CHRN subunits using the Single Cell-to-CT^TM^ Kit in 32 individual HBO cells. The final real-time PCR products were separated by electrophoresis on a 2% agarose gel containing 1 μg/ml ethidium bromide. In **(A)** T2R38 was not detected in cell number 1–11.

To further investigate the differential expression of CHRN subunits in individual cells, single cell PCR was performed to detect the expression of CHRNA5, CHRNA6, CHRNB4 and T2R38 mRNAs in 32 individual HBO cells (Fig 11A–11C). GAPDH was used as a control (Fig 11A). We did not observed T2R38 mRNA expression in cell number 1–11 (Fig 11A). Out of 32 HBO cells examined, 14 cells (43.7%) were positive for one or more CHRN mRNAs, 7 cells (21.9%) expressed multiple CHRNs, and 6 cells (18.7%) were positive for T2R38 mRNA (Fig 11A–11C). Out of 6 T2R38 positive cells, 3 cells (50%) co-expressed CHRNs (Fig 11A and 11B). These results suggest that CHRNs are expressed in a subset of T2R38 positive cells.

### Co-immunoprecipitation (co-lP) of CHRN subunits in HBO cells

**Fig 12:**
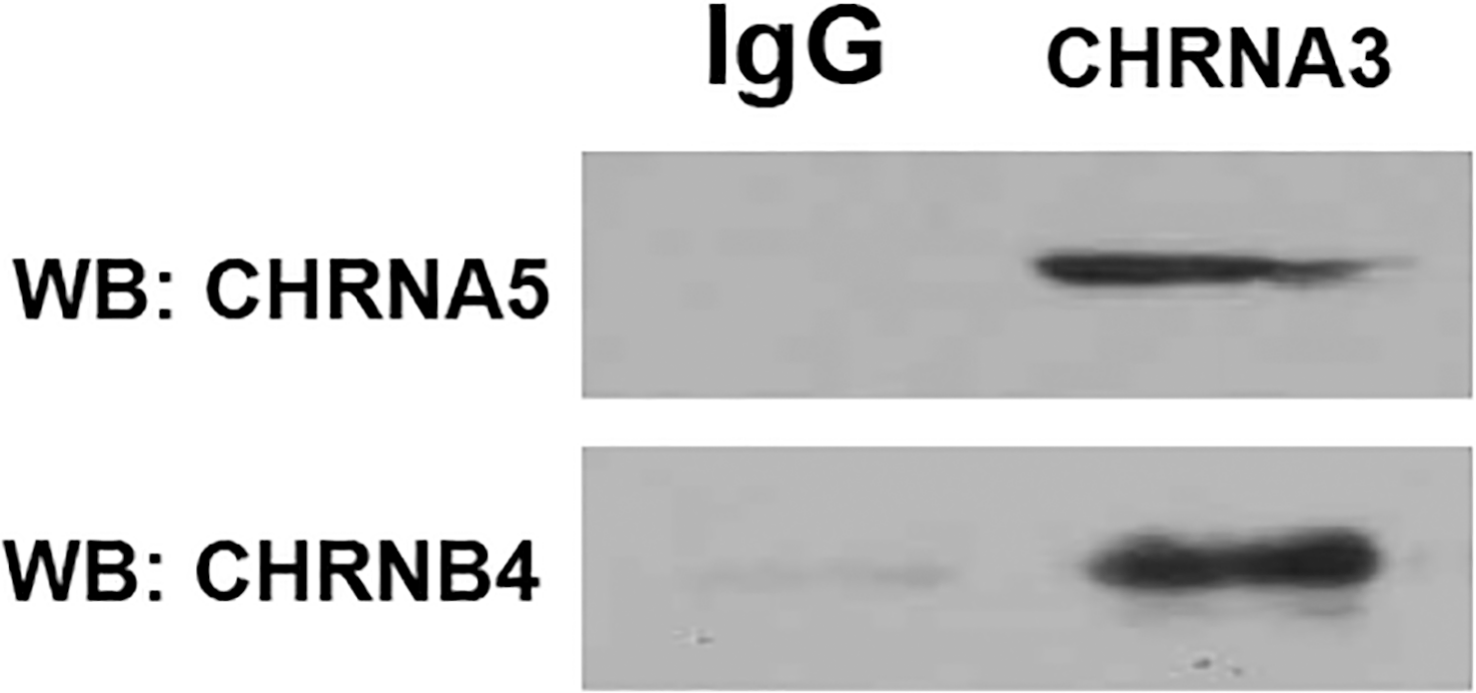
Co-IP of CHRNs in HBO cell lysates. In HBO cell lysates, CHRNA5 and CHRNB4 proteins were immune-precipitated by AChRα3 antibody. WB = Western blot; IgG = negative control.

To investigate if multiple CHRN subunits combine to form complex CHRNs in HBO cells, we performed co-IP studies in HBO cell lysates. In HBO cell lysates, the AChRα3 antibody immune-precipitated CHRNA5 and CHRNB4 proteins (Fig 12). These results suggest that in HBO cells CHRN subunits can assemble to form complex CHRNs containing CHRNA3, CHRNA5 and CHRNB4 subunits and perhaps other subunits.

### Effect of acute Nic and ETOH exposure on CHRN mRNA levels in HBO cells

**Fig 13:**
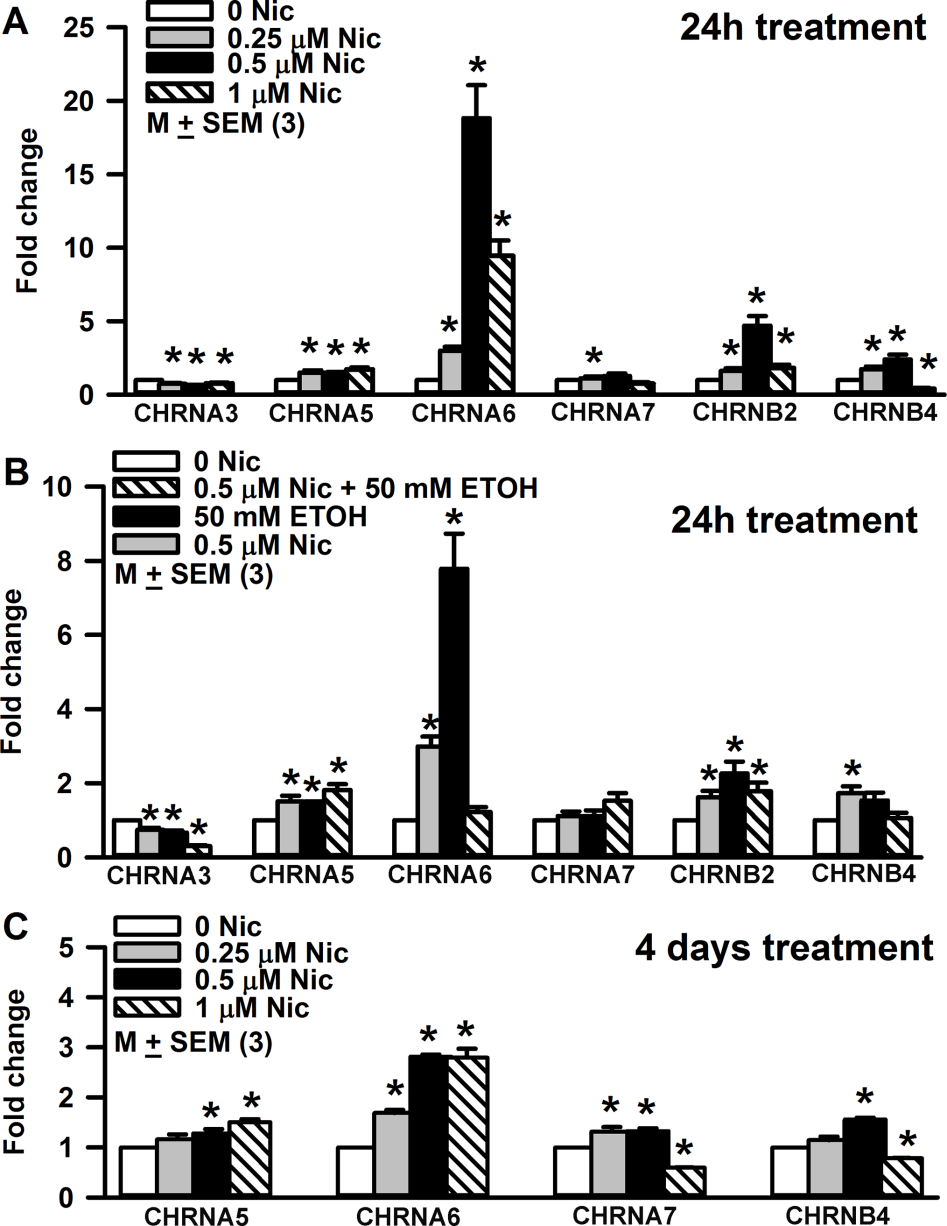
Effect of Nic and ETOH treatment on CHRNA5 and CHRNA6 protein expression in HBO cells. HBO cells were treated with Nic (0.25–1.0 μM), ETOH (50 mM), and 0.5 μM Nic + 50 mM ETOH for 24h. Western blots were developed using specific AChRα5 or AChRα6 antibodies **(A)** and analyzed **(B and C)**. The fold change in protein expression was calculated with respect to β-actin. The values are means of triplicate measurements.

As described in detail previously [14], treating STC-1 cells with Nic induced changes in the expression of CHRNA4, CHRNA5, CHRNA6, and CHRNB4 mRNAs with only minor changes in the mRNA levels of PLCβ2 and TRPM5. In mice, chronic exposure to Nic or ETOH also induced significant changes in CHRN mRNAs in CV taste bud cells [5]. In alcohol non-preferring rats, chronic exposure to 5% ETOH produced a 9 fold increase in CHRNA5 mRNA expression with only minor changes in the mRNA expression of T1R3, T2R38 and TRPM5 [16]. Taken together, these results indicate that CHRNs expressed in taste cells or enteroendocrine cells of the gut are a major target of Nic and ETOH. Accordingly to investigate the effect of acute Nic treatment on CHRN mRNA expression levels, HBO cells were treated with 0.25 μM, 0.5 μM or 1.0 μM Nic for 24 h (Fig 13A). Relative to control, increasing Nic concentration from 0.25 to 0.5 μM, induced a dose dependent increase in CHRNA6, CHRNB2, and CHRNB4 mRNA levels. However, at 1.0 μM Nic, CHRNA6, CHRNB2, and CHRNB4 mRNA levels were significantly lower than the levels observed at 0.5 μM Nic (Fig 13A). While increasing Nic concentrations induced a decrease in the CHRNA3 mRNA levels, no significant changes were observed in CHRNA7 mRNA levels after acute Nic treatment. These results indicate that Nic differentially affects the expression of CHRN mRNAs in HBO cells in a dose dependent manner.

Relative to control, treating HBO cells with ETOH (50 mM) induced an increase in CHRNA5, CHRNA6, and CHRNB2 mRNA levels (Fig 13B). These changes were accompanied by a decrease in CHRNA3 mRNA level. Interestingly, when HBO cells were treated with a mixture containing 50 mM ETOH + 0.5 μM Nic, there was a significant decrease in the CHRNA3 (p = 0.0049) and CHRNA6 (p = 0.0024) mRNA levels relative to their levels in the presence of 50 mM ETOH alone (Fig 13B). In the presence of the mixture, mRNA levels for CHRNA3 (p = 0.0023) and CHRNA6 (0.0042) were also lower than those observed in the presence of 0.5 μM Nic alone (Fig 13B). These results indicate that treating HBO cells with a mixture containing Nic + ETOH inhibits CHRNA3 and CHRNA6 mRNA expression in HBO cells. It is important to note that CHRNA7 mRNA levels in HBO cells were not altered by either Nic or ETOH treatment. These results further demonstrate that both Nic and ETOH produce major effects on CHRNA6 mRNA expression.

To investigate the effect of chronic Nic treatment on CHRN mRNA expression, HBO cells were treated with 0.25 μM, 0.5 μM or 1.0 μM Nic for 4 days (Fig 13C). Relative to control, at 0.5 μM Nic, a significant increase was observed in CHRNA5, CHRNA6, CHRNA7, and CHRNB4 mRNA levels (Fig 13C). Relative to 0.5 μM Nic, at 1.0 μM Nic CHRNA7 and CHRNB4 mRNA levels were significantly decreased. Thus, unlike the condition with acute Nic treatment, chronic Nic treatment induced significant changes in CHRNA7 mRNA levels.

### Effect of acute Nic and ETOH exposure on CHRN protein levels in HBO cells

**Fig 14:**
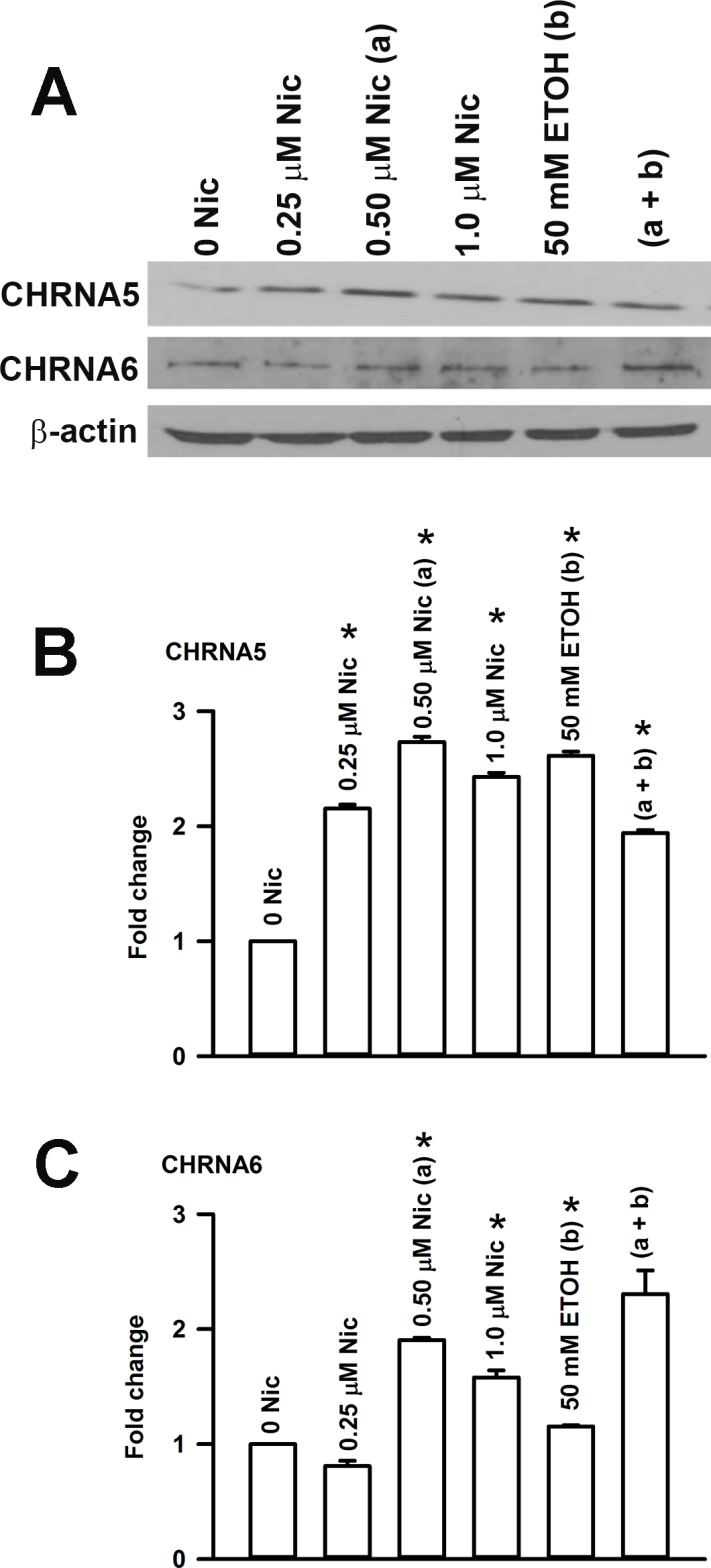
Effect of Nic exposure on the CHRN subunit mRNA expression level in HBO cells. **(A) After 24h Nic treatment**. Relative to control, at 0.25 μM Nic the *p values for CHRNA3, CHRNA5, CHRNA6, CHRNB2, and CHRNB4 mRNAs were 0.0111, 0.0306, 0.0017, 0.0239, and 0.0173, respectively. Relative to control, at 0.50 μM Nic the *p values for CHRNA3, CHRNA5, CHRNA6, CHRNB2, and CHRNB4 mRNAs were 0.0022, 0.0473, 0.0014, 0.0049, and 0.0141, respectively. Relative to control, at 1.0 μM Nic the *p values for CHRNA3, CHRNA5, CHRNA6, CHRNB2, and CHRNB4 mRNAs were 0.0194, 0.0055, 0.0013, 0.0236, and 0.0003, respectively. Relative to 0.25 μM Nic, at 0.50 μM Nic the *p values for CHRNA6 and CHRNB4 were 0.0039 and 0.0024. Relative to 0.50 μM Nic at 1.0 μM Nic the *p values for CHRNA6, CHRNB2, and CHRNB4 were 0.0192, 0.0144, and 0.0046, respectively. **(B) After 24h ETOH treatment**. Relative to control, at 50 mM ETOH the *p values for CHRNA3, CHRNA5, CHRNA6, and CHRNB2 mRNAs were 0.0048, 0.0207, 0.0020, and 0.0169, respectively. Relative to 50 mM ETOH, at 50 mM ETOH + 0.5 μM Nic the *p values for CHRNA3 and CHRNA6 mRNAs were 0.0049 and 0.0024. **(C) After 4 days treatment**. Relative to control, at 0.25 μM Nic the *p values for chrna6 and CHRNA7 mRNAs were 0.0003 and 0.024, respectively. After 4 days treatment, relative to control, at 0.50 μM Nic the *p values for CHRNA5, CHRNA6, CHRNA7, and CHRNB4 were 0.0402, 0.0001, 0.0049, and 0.0001, respectively. Relative to control, at 1.0 μM Nic, the *p values for CHRNA5, CHRNA6, CHRNA7, and CHRNB4 were 0.0009, 0.0005, 0.0001, and 0.0001, respectively. The values represent mean ± SEM of triplicate measurements.

Western blots were analyzed for CHRNA5 and CHRNA6 protein expression using specific AChRα5 or AChRα6 antibody relative to β-actin (Fig 14A). At 0.25 μM and 0.5 μM Nic there was a dose-dependent increase in CHRNA5 protein expression (p = 0.0001) (Fig 14B). Increasing Nic to 1.0 μM produced a significant decrease in CHRNA5 protein relative to its level in 0.5 μM Nic (p = 0.0065). Relative to control, CHRNA5 protein expression demonstrated an increase (p = 0.0001) after 50 mM ETOH treatment (Fig 14B). However, CHRNA5 protein expression in the presence of 0.5 μM Nic + 50 mM ETOH mixture was significantly less than its level in 50 mM ETOH alone (p = 0.0001) or 0.5 μM Nic alone (p = 0.0001) (Fig 14B).

In another set of HBO cells, we detected a small decrease (p = 0.015) in CHRNA6 protein expression after 0.25 μM Nic exposure (Fig 14C). At 0.50 μM Nic, the CHRNA6 protein expression increased above control (p = 0.0001). At 1.0 μM Nic, the CHRNA6 protein expression decreased relative to 0.5 μM Nic (p = 0.0072). Relative to control, CHRNA6 protein expression demonstrated a small but significant (p = 0.0001) increase after 50 mM ETOH treatment. However, CHRNA6 protein expression in the presence of 0.5 μM Nic + 50 mM ETOH mixture was not statistically different from the level in the presence of 0.5 μM Nic alone (Fig 14C).

Taken together, the above results show that in HBO cells, acute and chronic exposure to Nic produces changes in CHRN mRNA and protein levels in a dose-dependent manner. In our earlier studies in STC-1 cells, acute and chronic exposure to Nic also produced dose-dependent changes in the CHRN mRNA levels [14].

### Effect of Nic on BDNF in HBO cells

**Fig 15:**
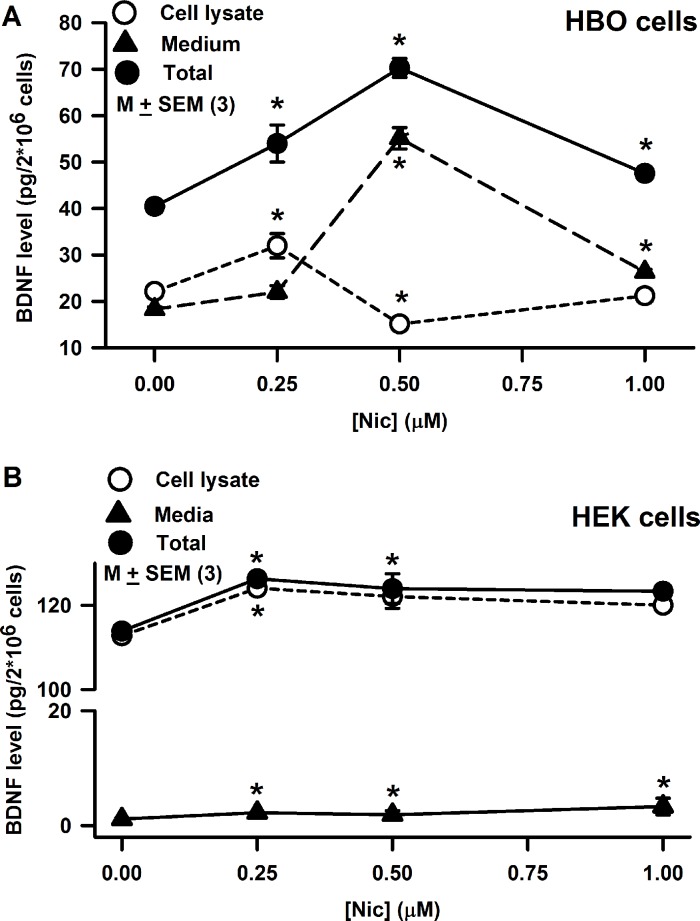
Effect of Nic on BDNF synthesis and release in HBO cells and HEK293 cells. **(A)** HBO cells were treated with 0.25, 0.50, and 1.0 μM Nic for 30 min. Following that BDNF concentration was measured in cell lysate and the media. Relative to control BDNF concentration in cell lysate increased at 0.25 μM Nic (p = 0.0274) and decreased at 0.5 μM Nic (p = 0.0055). No significant increase in BDNF concentration was observed at 0.25 μM Nic, however, at 0.5 μM Nic, BDNF concentration was significantly increased relative to control (p = 0.0001). Relative to control at 1.0 μM Nic, a small but significant increase in the BDNF concentration was observed in the media (p = 0.0004). The p values for the changes in total BDNF content for 0.25, 0.5 and 1.0 μM Nic were 0.0289, 0.0002, and 0.0014, respectively. The values are mean ± SEM of triplicate measurements. **(B)** In HEK293 cells, BDNF concentration in cell lysate was about 6 times greater than in HBO cells. Relative to control, at 0.25 μM Nic, BDNF concentration in cell lysate was significantly increased (p = 0.0001), and remained elevated at 0.5 μM (p = 0.0331) and 1.0 μM Nic (p = 0.0041). Relative to control at 0.25 μM, 0.5 μM, and 1.0 μM Nic, a small but significant increase in the BDNF concentration was observed in the media (p = 0.0001). The values are mean ± SEM of triplicate measurements.

HBO cells were treated with 0.25 μM, 0.5 μM or 1.0 μM Nic for 30 min. The total amount of BDNF in cell lysis plus in the medium increased with 0.25 μM (p = 0.0289) and 0.5 μM Nic (p = 0.0002) treatment and decreased at 1 μM Nic (p = 0.0014) (Fig 15A). When BDNF concentration was examined separately in the cell lysates and the media, BDNF content increased in the media at 0.5 μM Nic (p = 0.0001) with a concomitant decrease in the BDNF concentration in the cell lysate (p = 0.0004). However, no significant changes were observed in the BDNF concentration in the cell lysate or the media at 1 μM Nic (Fig 15A). These results suggest that the effect of Nic on BDNF synthesis and release in HBO cells is dose-dependent. We have previously shown that Nic exposure in STC-1 cells decreases BDNF concentration in cell lysates [14]. Exposure of neonate rats to Nic has been shown to cause a decrease in the expression of nerve growth factor and BDNF in hippocampus and frontal cortex [17].

### Effect of Nic on BDNF in HEK293 cells

In contrast to HBO cells, in HEK293 cells the BDNF concentration in cell lysate was about 6 times higher (Fig 15B). Treating HEK293 cells with 0.25 μM Nic, significantly increased the content in cell lysate that remained elevated at 0.5 and 1.0 μM Nic. Relative to control, treating HEK293 cells with 0.25, 0.5 and 1.0 μM Nic produced a small but dose dependent increase in BDNF concentration in the media (Fig 15B). These results suggest that although the intracellular concentration of BDNF is significantly higher than in HBO cells, the release of BDNF from HEK293 cells in the presence of Nic is significantly smaller.

### Effect of acute Nic exposure on HBO cell Ca^2+^ ([Ca^2+^]_i_)

**Fig 16:**
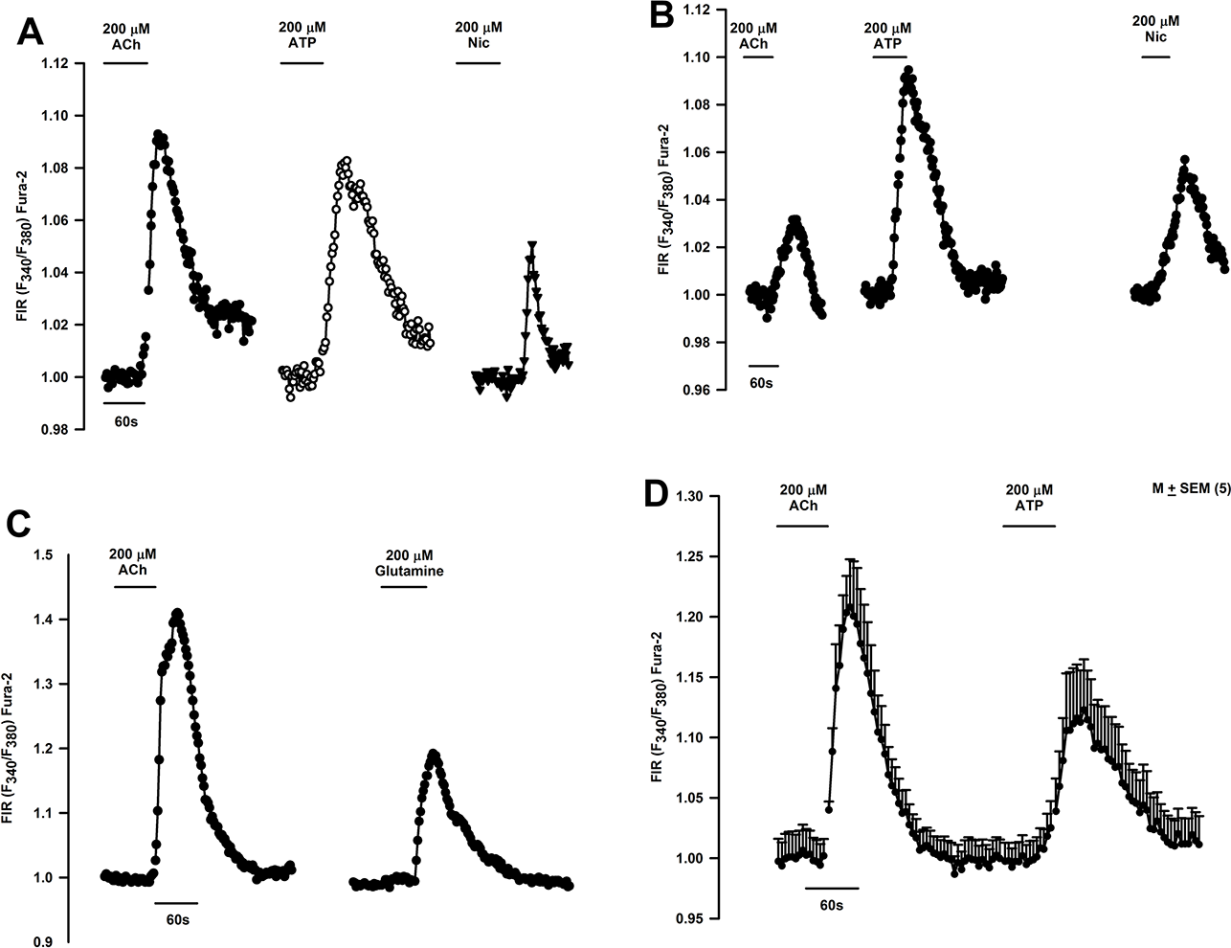
Effect of Nic, acetylcholine (ACh), and other taste stimuli on temporal changes in [Ca^2+^]_i_ in individual HBO cells. Temporal changes in [Ca^2+^]_i_ were monitored as changes in Florescence Intensity Ratio (FIR; F340/F380) in Fura-2 loaded single HBO cells in response to stimulation with different taste stimuli. **(A and B)** Show two representative HBO cells that responded with a transient increase in FIR when stimulated with 200 μM ACh, 200 μM ATP, and 200 μM Nic. **(C)** Shows another HBO cell that responded with a transient increase in FIR when stimulated with 200 μM ACh and 200 μM glutamine. **(D)** Shows mean ± SEM changes in FIR from 5 individual HBO cells that were stimulated with 200 μM ACh and 200 μM ATP. In each cell, the FIR was normalized to 1 with respect to its value under the control conditions.

Only a subset of HBO cells express CHRNs (Figs 2–9). We hypothesize that Nic, a bitter stimulus, will induce an increase in [Ca^2+^]_i_ in a subset of HBO cells by interacting with both T2Rs and CHRNs. The increase in [Ca^2+^]_i_ due to the interaction of Nic with CHRNs should be inhibited by Mec. Accordingly, we monitored changes in [Ca^2+^]_i_ as temporal changes in FIR (F_340_/F_380_) in individual HBO cells loaded with Fura-2 as shown in Panel A in S1 Fig (HBO cells). Nic evoked a transient increase in [Ca^2+^]_i_ in individual HBO cells (Fig 16A and 16B). The cells that responded to Nic also responded to ACh and ATP (Fig 16A and 16B). An additional HBO cell that responded to ACh also responded to glutamine (Fig 16C). Fig 16D shows mean changes in FIR in response to ACh and ATP in 5 individual HBO cells.

**Fig 17:**
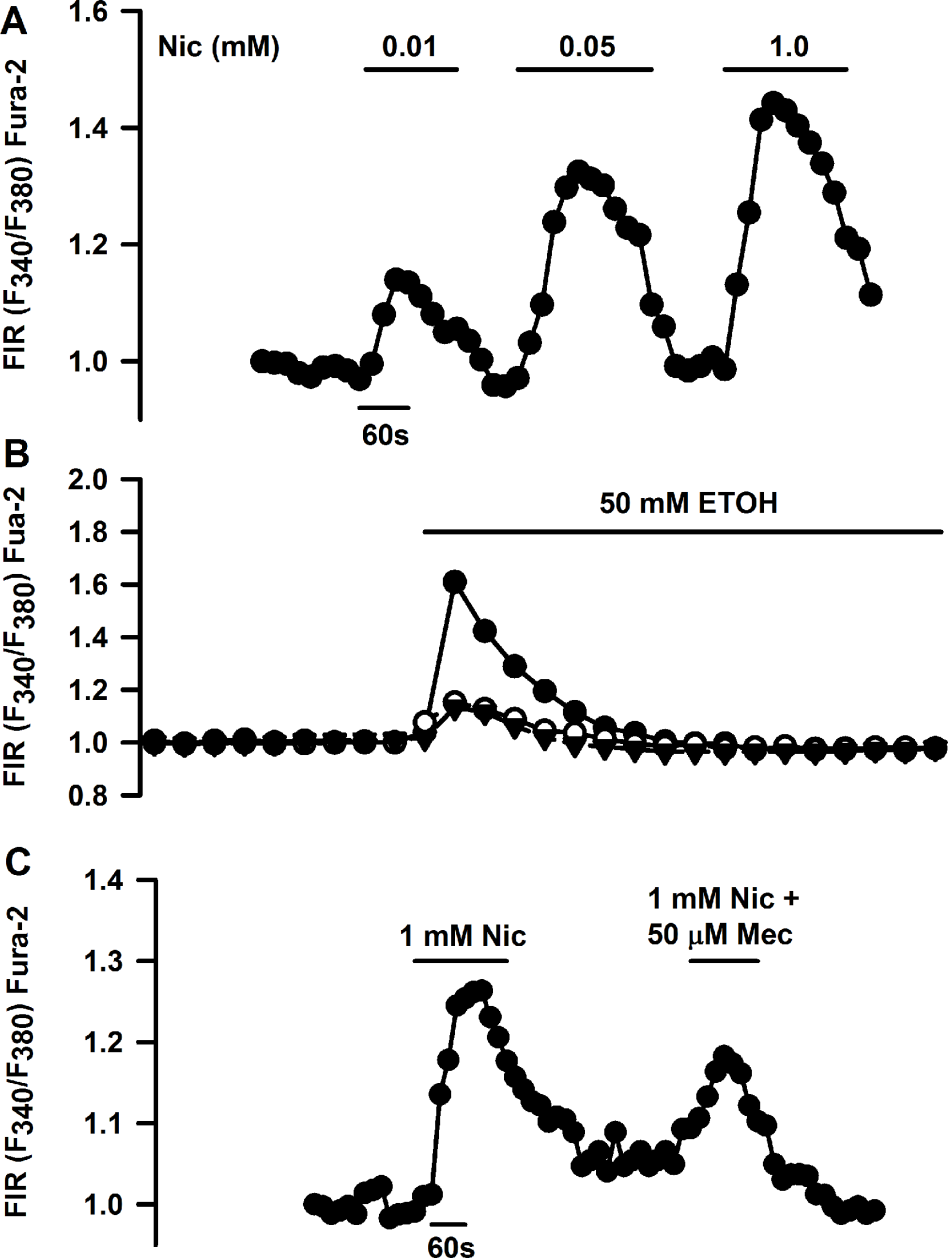
Effect of Nic, ETOH, and mecamylamine (Mec) on temporal changes in [Ca^2+^]_i_ in individual HBO cells. **(A)** Shows a representative HBO cell that responded with a dose-dependent increase in FIR when stimulated with 0.01, 0.05 and 1.0 mM Nic. **(B)** Shows 1 out of 8 HBO cells that responded with a transient increase in FIR when stimulated with 50 mM ETOH. Two additional cells responded with a small but significant increase in FIR when stimulated with 50 mM ETOH. **(C)** Shows another representative HBO cell that responded with an increase in FIR in the presence of 1 mM Nic. Mec (50 μM) inhibited the Nic-induced increase in FIR. In each cell, the FIR was normalized to 1 with respect to its value under the control conditions.

Fig 17A shows a representative HBO cell that responded with a dose-dependent increase in FIR when stimulated with 0.01, 0.05 and 1.0 mM Nic. In 1 out of 8 cells investigate, one cell responded with a robust increase in [Ca^2+^]_i_ after exposure to 50 mM ETOH. Two additional cells gave a modest increase in [Ca^2+^]_i_ after ETOH stimulation (Fig 17B). Fig 17C shows another representative HBO cell that responded to 1 mM Nic. The Nic-induced transient increase in [Ca^2+^]_i_ was significantly inhibited in the presence of 50 μM Mec. These studies indicate that HBO cells express functional CHRNs that form Mec-sensitive cation channels.

### Effect of acute Nic exposure on HEK293 cell [Ca^2+^]_i_

**Fig 18:**
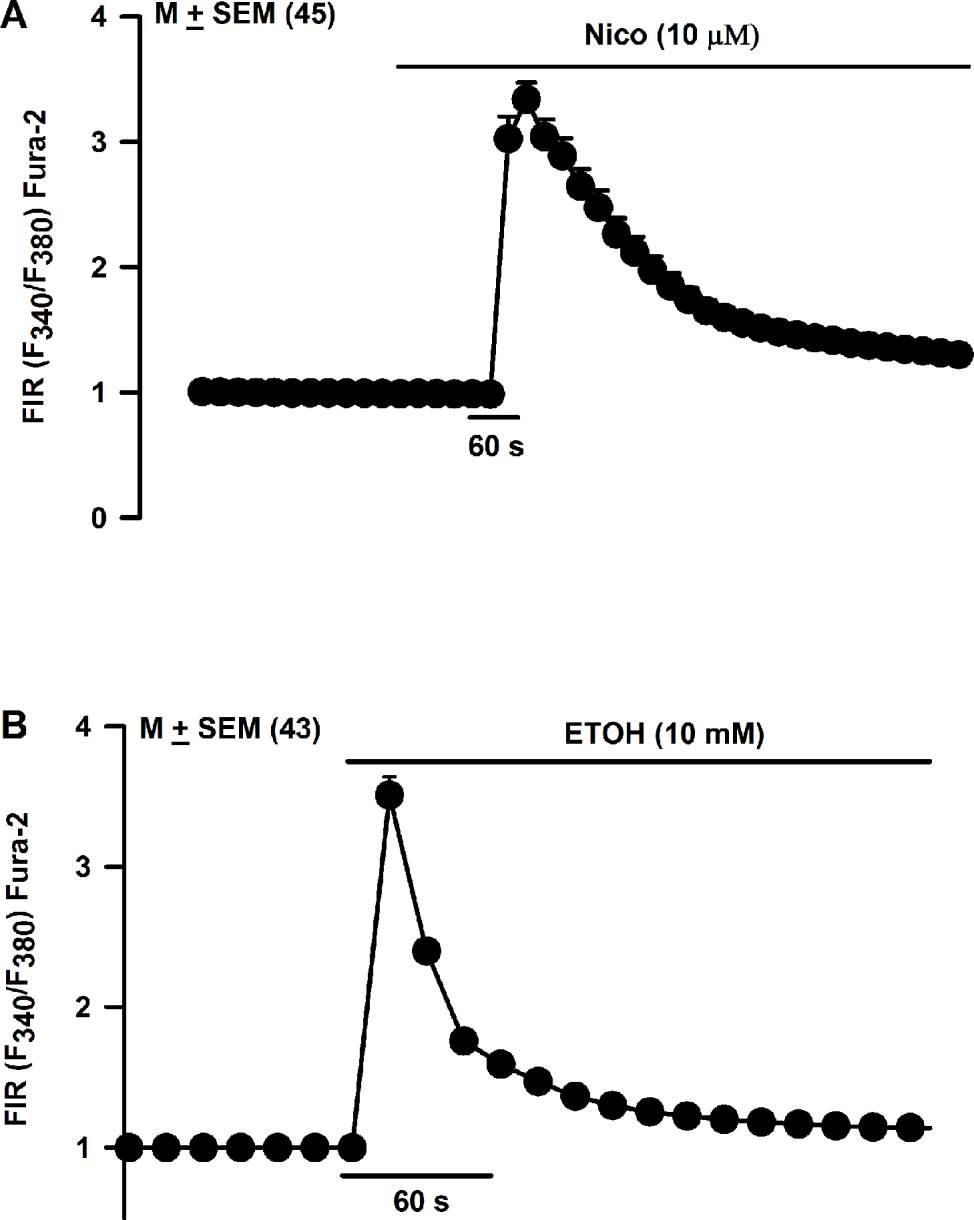
Effect of Nic and ETOH on temporal changes in [Ca^2+^]_i_ in individual HEK293 cells. **(A)** Shows changes in FIR in all 45 HEK293 cells in the visual field that were stimulated with 0.01 mM Nic. In each cell, the FIR was normalized to 1 with respect to its value under the control conditions. The values are mean ± SEM of the number of cells in the visual field. **(B)** Shows changes in FIR in all 43 HEK293 cells in the visual field that were stimulated with 10 mM ETOH. In each cell, the FIR was normalized to 1 with respect to its value under the control conditions. The values are mean ± SEM of the number of cells in the visual field.

In contrast to HBO cells, all 45 HEK293 cells loaded with Fura-2 as shown in Panel B in S1 Fig (HEK cells) responded with a robust increase in [Ca^2+^]_i_ upon stimulation with 10 μM Nic (Fig 18A). In addition, all 43 HEK293 cells responded with a robust increase in [Ca^2+^]_i_ upon stimulation with 10 mM ETOH (Fig 18B). These results are consistent with our IHC studies that show a much higher and wider distribution of CHRNs in HEK cells relative to HBO cells.

## Discussion

### CHRN expression in HBO cells

CHRNs have been shown to be expressed in rodent CV and FF TRCs [5], STC-1 cells [14], enteroendocrine cells of the gut [5, 14], and in epithelial cells of intrapulmonary airways that also co-express TRPM5 [18]. However, at present the information regarding CHRN expression and function in human taste cells is lacking. In a stably proliferating human taste cell line (HTC-8), RT-PCR technique only demonstrated the gene expression of CHRNA5 [6]. It is not clear if HTC-8 cells do not express additional CHRNs or their expression levels are too low for detection.

In this paper we used HBO cells to investigate the expression and function of CHRNs. Parallel studies on CHRN expression and function were carried out in HEK293 cells, an epithelial cell line that has been shown to endogenously express CHRNA7 and CHRNA5 [10]. At present a detailed investigation of endogenous CHRN expression and function in HEK293 cells is also lacking. Parallel studies on HBO cells and HEK293 cells allowed us do a comparative analysis of CHRN expression and function in these two cell lines of human origin.

Similar to the case in HTC-8 cells [6], we detected the expression of CHRNA5 mRNA (Fig 1A) and protein (Fig 1C) in HBO cells. In contrast to HTC-8 cells, we also detected the mRNAs for CHRNA3, CHRNA4, CHRNA6, CHRNA7, CHRNB2, CHRNB4 (Fig 1A), and CHRNA4 protein in HBO cell lysate (Fig 1C). Both HTC-8 cells [6] and HBO cells demonstrate the expression of mRNAs for T1R1, T1R3, T2R38, PLCβ2, and TRPM5 (Fig 1B). We have previously demonstrated the presence of all four (α, β, γ, and δ) ENaC subunits in a subset of HBO cells [9].

Further confirmation of CHRN expression in HBO cells was obtained by using specific antibodies to various CHRNA and CHRNB subunits. In our ICC studies, CHRNA3, CHRNA4, CHRNA5, CHRNA6, CHRNA7, CHRNB2, CHRNB4 were expressed in a subset of HBO cells (Figs 2–9). CHRNA5 was co-expressed in HBO cells with CHRNA3 and CHRNA4 (Fig 3), and with CHRNB2 (Fig 4C and 4D). In addition, CHRNA3 was co-expressed in HBO cells with CHRNB3 (Fig 4A and 4B). In single cell PCR studies, 43.7% of HBO cells expressed one or more CHRN mRNAs and 21.9% of HBO cells demonstrated the expression of multiple CHRNs (Fig 11).

CHRNA4 (Fig 6A), CHRNA5 (Fig 5A and 5B), CHRNAA6 (Fig 7) and CHRNB2 (Fig 6B) were expressed in cells that were positive for TRPM5. These results are consistent with the expression of CHRNs in TRPM5 positive mouse CV and FF taste bud cells [5]. In our ICC studies CHRNA5 was also co-localized in a subset of HBO cell that were positive for T1R3 (Fig 8) and CHRNB2 was co-localized in a subset of HBO cells that were positive for T2R38 (Fig 9). In our single cell PCR experiments 50% of the T2R38 positive cells also expressed one or more CHRNs (Fig 11). Taken together, our ICC and single cell PCR studies suggest that CHRNs are expressed in TRPM5 positive cells that are either T2R38 positive or T1R3 positive. The expression of CHRN and markers of taste cells detected by RT-PCR, qRT-PCR, ICC, immunohistochemistry (IHC) and *in situ* hybridization (ISH) in HBO cells, HEK293 cells, STC-1 cells [14], and mouse taste receptor cells (TRCs) [5] are summarized in S1 Table. Thus, in HBO cells, STC-1 cells, HEK293 cells and mouse TRCs, CHRNs localize in TRPM5 positive cells. Currently, the role of CHRNs in T1R3 expressing cells is not clear. However, the expression of CHRNs in T2R38 positive (bitter sensing) cells is consistent with the involvement of CHRNs in the neural and behavioral responses to Nic and ETOH in rodents [2, 4, 5]. In future studies, single cell PCR analysis for all CHRNA and CHRNB subunits in a much larger population of HBO cells will give a clearer picture of CHRN expression in HBO cells.

Individual HBO cells responded with a transient increase in [Ca^2+^]_i_ when stimulated with Nic or ACh (Figs 16 and 17). Individual HBO cells demonstrated a dose-dependent increase in [Ca^2+^]_i_ when stimulated with increasing concentration of Nic (Fig 17A). Nic effects on [Ca^2+^]_i_ were partially Mec-sensitive (Fig 17C). Individual HBO cells also responded to ETOH stimulation with a transient increase in [Ca^2+^]_i_ (Fig 17B). Muscarinic acetylcholine receptors have been shown to be expressed in TRCs [19, 20]. Inhibition of Nic-induced increase in [Ca^2+^]_i_ by Mec (Fig 17C) indicates that in addition to muscarinic acetylcholine receptors, CHRNs are expressed in a subset of HBO cells. Collectively, the above data demonstrate that a subset of HBO cells express functional CHRN receptors. As described in detail previously [4], in our whole cell patch clamp studies, Nic elicited currents in a subset of isolated rat FF TRCs that were inhibited in the presence of 0.3 mM Mec. Although most of the current in CHRNs is carried by Na^+^ and K^+^, Ca^2+^ make a significant contribution [21]. Activation of CHRNs causes cell depolarization that activates voltage-gated ion channels and allows Ca^2+^ influx. However, our data indicates that in HBO cells CHRNs, by themselves, serve as the Ca^2+^ entry pathway (Fig 17C). The higher Ca^2+^ permeability of the α7 CHRN is due to the arrangements of charged residues at the inner mouth of the ionic pore and polar residues in the outer part of the channel [21].

Individual HBO cells demonstrate differential expression of CHRNs (Fig 11) and can form complex receptors comprising multiple subunits (Fig 12). While CHRNA7 subunits assemble as homopentamers, most of the other CHRNs assemble into heteropentamers comprising two or more different kinds of subunits. The α2, α3, α4 or α6 subunits may combine in a binary fashion with β2 or β4 subunits. These complexes give rise to ligand binding and/or functional CHRNs. The β3 and α5 subunits are termed as ‘wild cards” and can integrate into complexes with two other subunits to form more complex receptors [22]. CHRNs that are most abundantly detected in brain are homomeric α7 and α4β2 [23]. The heteromeric α4β2 subtypes demonstrates high affinity for Nic and the homomeric α7 provides α-bungarotoxin-binding sites in the brain [21]. However, α4β2* CHRNs can also exist in several isoforms forms: (α4)_2_(β2)_3_ or (α4)_3_(β2)_2_ that show different sensitivities towards Nic [22]. In addition, there is evidence to show that α7 and β2 CHRNs can assemble to form heteromeric functional channels [24].

Genetic association studies in humans show that gene variants in CHRNA5 influence both ETOH and Nic dependence [24–28]. The α5 subunit functions as an accessory subunit and is only co-expressed with other α and β CHRN subunits. It facilitates α4* CHRNs assembly *in vitro*. The α5 subunit is critical for controlling the expression and functional role of a population of α4*-containing CHRNs in the ventral tegmental area (VTA) [29].

The α6 subunit is expressed in midbrain dopaminergic regions associated with pleasure, reward, and mood control. This suggests that α6*-containing CHRNs play an important role in Nic dependence and in Nic induced changes in mood and emotions [22]. The dopaminergic neurons from the posterior VTA have been reported to express α6 and β3 subunits with the α4 and β2 subunits [30, 31]. VTA provides main projection to the nucleus accumbens. However, at present the role of complex CHRNs, expressed in the taste bud cells, in the detection of the bitter taste of Nic and ETOH is not clear. To further clarify the role of individual CHRNs in the detection of the bitter taste of Nic and ETOH, in future studies, we will monitor CT responses to Nic and ETOH in specific CHRN KO mice.

### CHRNs and BDNF regulation

CHRNs have been shown to be involved in structuring and maintenance of neurites and synapses, modulation of neuronal viability and death, and in the control of transmitter release [22; 32]. Our results suggest a potential role of CHRNs in the regulation of BDNF synthesis and release in HBO cells (Fig 15A). In the taste system, BDNF and its cognate receptor trkB are present mainly in type III taste cells [33, 34] and are required for the developmental remodeling of taste bud innervation [35] and to maintain normal amounts of innervation to adult taste buds [36–38]. Exposure of neonate rats to Nic causes a decrease in the expression of nerve growth factor and BDNF and affects both brain development and impairs brain function [17]. In addition, BDNF is present in the gut. It participates in survival and growth of enteric neurons, augmentation of enteric circuits, and stimulation of intestinal peristalsis and propulsion [15, 39].

### Upregulation of CHRNs in HBO cells by Nic and ETOH

Smokers have an average serum concentration of 100–200 nM Nic after smoking [40, 41]. In our studies, exposing HBO cells acutely (24h) to 250–500 nM Nic concentrations significantly enhanced mRNAs for CHRNA5, CHRNA6, CHRNB2 and CHRNB4. At 1.0 μM Nic, the CHRNA6, CHRNB2, and CHRNB4 mRNA levels were significantly lower than the levels observed at 0.5 μM Nic (Fig 13A).

Similar changes in CHRN mRNA and protein levels have been observed in other cells. Treating human dermal fibroblasts with 10 μM nicotine for 24h produced 1.8 to 3.8 fold increase in the expression of CHRNA3, CHRNA5, CHRNA7, CHRNB2, and CHRNB4. These changes were not observed in the presence of Mec [42]. In human squamous cell lung cancer (SCC-L) cell lines and SCC-L tumors, 100 nM Nic increased the levels of α7-nAChR mRNA and α7-nAChR transcription [43]. In K-177, a stable cell line (HEK293) expressing human CHRNA4 and CHRNB2, Nic (100 nM) caused upregulation of the α4β2 receptor [44]. Similar effects were reported at 1 μM Nic [45].

After chronic (4 days) Nic (0.5 μM) exposure, a significant increase in the CHRNA5, CHRNA6, CHRNA7, and CHRNB4 mRNA levels was observed (Fig 13C). Relative to 0.5 μM Nic, at 1.0 μM Nic the CHRNA7 and CHRNB4 mRNA levels were significantly decreased. Thus, unlike the condition with acute Nic treatment, chronic Nic treatment induced significant changes in CHRNA7 mRNA levels. In male rats continually self-administering Nic (approximately 1.5 mg free base/kg/day) was associated with upregulation of brain α4, α6, and β2 CHRN subunits [46]. Under chronic Nic exposure, α4β2 CHRNs undergo a Nic-induced upregulation of receptor numbers at the membrane in several areas of the brain [47]. Overexpressing CHRNs in mice increases sensitivity to Nic. The CHRNs comprising α5, α6, β3, and β4 subunits regulate Nic intake [48].

Exposure to Nic alters the trafficking and assembly of CHRNs, leading to their up-regulation in the plasma membrane [49]. Nic and cotinine increased the number of α4β2 receptors on the plasma membrane and caused a redistribution of intracellular receptors. In contrast to this, cotinine exposure down-regulated α6β2β3 receptors. Cotinine and Nic both alter the assembly of α4β2 receptors to favor the high sensitivity (α4)_2_(β2)_3_ stoichiometry [49]. Upregulation of α7 is systematically observed after incubation of lymphocytes with Nic or α-bungarotoxin [50]. Taken together, Nic exposure produces differential effects on the expression of CHRN mRNAs depending upon the dose, exposure time, and cell type.

Acute ETOH exposure also significantly upregulated CHRNA5, CHRNA6, and CHRNB2 but decreased the mRNA expression of CHRNA3 (Fig 13B). Polymorphisms localized to chromosome 15q24 that encodes α5, α6, β3, and β4 CHRNs are also linked to alcohol dependence and early initiation of alcohol use [48]. Over expression of CHRNs α5, α3, and β4 subunit genes reduces ETOH intake in mice [51].

In our studies, acutely exposing HBO cells to a mixture containing 0.5 μM Nic + 50 mM ETOH inhibited the increase in CHRNA6 mRNA observed with 0.5 μM Nic or 50 mM ETOH alone (Fig 13B). In rats with moderate ETOH preference, ETOH consumption increases after Nic treatment [52] and application of a CHRN blocker reduces ETOH intake [53]. Varenicline, a partial agonist of α4β2 CHRN subtype, used for cessation of smoking may also reduce alcohol intake [54]. This suggests that Nic and ETOH can interact on common CHRNs [48].

However, in some cases, there was no correlation between CHRN subunit protein and mRNA. In the temporal cortex of smokers there was an increase in the α4 and α7 CHRN subunit protein compared to non-smokers but there was no difference in CHRNA4 and CHRNA7 mRNA [55]. In some cultured human cell lines changes in CHRN numbers were not attributable to changes in CHRN mRNAs [56]. In these cases the Nic-induced upregulation of CHRN numbers is independent of transcriptional events [47].

As described in detail previously [14], we investigated the effect of 0.25, 0.5, and 1.0 μM Nic exposure for 24h on the differential expression of CHRN mRNAs in STC-1 cells. As summarized in S2 Table, in both HBO cells and STC-1 cells, Nic at 0.25 and 0.5 μM induced an increase in CHRNA5, CHRNA6, CHRNB2 and CHRNB4 mRNAs in a dose-dependent manner. Although at 1 μM Nic the enhancement was smaller than at 0.5 μM Nic, Nic still produced enhancement in CHRNA5, CHRNA6 and CHRNB2 mRNA relative to control (0 Nic). CHRNB4 mRNA increased in STC-1 cells but decreased in HBO cells. In contrast, in HBO cells and STC-1 cells, CHRNA3 and CHRNA7 mRNA expression did not change in the same direction in the presence of Nic. After 4 days of Nic treatment, Nic produced an increase in CHRNA5 and CHRNA6 mRNAs in HBO cells and STC-1 cells. These results suggest that in human TRCs and mouse enteroendocrine cells Nic consistently enhances the expression of CHRNA5 and CHRNA6 mRNAs. In the present study we did not test the effect of Nic or ETOH on G-protein coupled sweet, umami, and bitter taste receptors and their downstream intracellular signaling effectors.

### Upregulation of CHRNs in HEK293 cells by Nic

Changes in CHRN subunit expression following acute or chronic Nic or ETOH exposure was not monitored in HEK293 cells in this study. In HEK293 cells, that expressed heterologous α4β2 CHRNs, a significant increase in [^3^H]cytisine binding was observed when the cells were exposed to 100 nM Nic. A maximal 15-fold increase in binding was observed at 10 μM of Nic, consistent with the upregulation of CHRNs [57]. In HEK293 cells stably expressing α4β2 CHRNs, Nic markedly increased the density of the β2 subunit protein in cell membranes. This increase was proportional to the increase in CHRN binding sites [58]. Taken together, these results indicate that Nic and ETOH induce upregulation of functional CHRNs in both HBO cells and HEK293 cells.

In summary our data indicates that both HBO cells and HEK293 cells express endogenous CHRNs. In HBO cells CHRNs are involved in detecting the bitter taste of Nic and ETOH and in the release of BDNF.

## Supporting information

S1 Table Expression of CHRNs and markers in HBO cells, STC-1 cells, HEK293 cells and mouse TRCs. (DOCX)

S2 Table Nic-induced changes in CHRN mRNA expression in HBO and STC-1 cells. (DOCX)

S1 Fig Fura-2 loading in HBO cells and HEK293 cells. HBO and HEK293 cells were loaded with Fura-2. **(A)** Shows images of Fura-2 loaded HBO cells using excitation wavelengths of 340 and 380 nm. **(B)** Shows images of Fura-2 loaded HEK293 cells using excitation wavelengths of 340 and 380 nm.
(TIF)
