# Supplementary material for: Nicotinic acetylcholine receptor (CHRN) expression and function in cultured human adult fungiform (HBO) taste cells
Source: PLoS One. 2018 Mar 7;13(3):e0194089. doi: 10.1371/journal.pone.0194089 (PMC5841828; doi:10.1371/journal.pone.0194089)
Supplement: S1 Table — (DOCX) [file pone.0194089.s001.docx]

**S1 Table**

**Expression of CHRNs and markers in HBO cells, STC-1 cells, HEK293 cells and mouse TRCs**

|  | **HBO**  **cells** | | | **^#^STC-1**  **cells** | | | **HEK293**  **Cells** | | **^##^Mouse**  **TRCs** | |
| --- | --- | --- | --- | --- | --- | --- | --- | --- | --- | --- |
|  | **RT-**  **PCR** | **ICC** | **q-**  **PCR** | **RT-**  **PCR** | **ICC** | **q-**  **PCR** | **RT-**  **PCR** | **ICC** | **ISH** | **IHC** |
| **CHRNA3** | + | + | + | + | + | + | + | + |  | + |
| **CHRNA4** | + | + |  | + | + | + | + | + |  | + |
| **CHRNA5** | + | + | + | + | + | + | + | + |  |  |
| **CHRNA6** | + | + | + | + |  | + | + |  |  |  |
| **CHRNA7** | + |  | + | + | + | + |  |  | + | + |
| **CHRNB2** | + | + | + | + | + | + | + | + | + | + |
| **CHRNB4** | + | + | + | + | + | + | + |  | + | + |
| **T1R1** | + |  |  |  |  |  |  |  |  |  |
| **T1R3** | + | + |  |  |  |  |  |  |  |  |
| **T2R38** | + | + |  | + |  |  | + |  |  |  |
| **TRPM5** | + | + |  | + |  | + | + |  | + | + |
| **PLCβ2** | + |  |  |  |  | + |  |  |  |  |

TRCs = Taste receptor cells; ICC = immunocytochemistry; ISH = *in situ* hybridization; IHC = immunohistochemistry

^#^Original data is presented in reference [14]

^##^Original data is presented in reference [5]
