# Supplementary material for: Nicotinic acetylcholine receptor (CHRN) expression and function in cultured human adult fungiform (HBO) taste cells
Source: PLoS One. 2018 Mar 7;13(3):e0194089. doi: 10.1371/journal.pone.0194089 (PMC5841828; doi:10.1371/journal.pone.0194089)
Supplement: S2 Table — (DOCX) [file pone.0194089.s002.docx]

**S2 Table**

**Nic-induced changes in CHRN mRNA expression in HBO and STC-1 cells**

| **CHRN** | **Nic (μM)** | **HBO (24h)** | **STC-1 (24h)** | **HBO (4d)** | **STC-1 (4d)** |
| --- | --- | --- | --- | --- | --- |
| **CHRNA3** | 0.25 | 0.742 | 1.017 |  | 1.315 |
| **CHRNA5** | 0.25 | **1.506** | **3.098** | 1.164 | **3.055** |
| **CHRNA6** | 0.25 | **2.989** | **5.005** | **1.688** | **3.985** |
| **CHRNA7** | 0.25 | 1.115 | 0.766 | 1.316 | 0.905 |
| **CHRNB2** | 0.25 | **1.618** | **1.855** |  | **1.855** |
| **CHRNB4** | 0.25 | **1.729** | **1.444** | 1.144 | **2.761** |
| **CHRNA3** | 0.50 | 0.613 | 1.143 |  | 1.010 |
| **CHRNA5** | 0.50 | **1.407** | **9.557** | **1.274** | **2.960** |
| **CHRNA6** | 0.50 | **18.821** | **13.225** | **2.806** | **4.555** |
| **CHRNA7** | 0.50 | 1.268 | 1.370 | 1.322 | 0.434 |
| **CHRNB2** | 0.50 | **4.696** | **2.601** |  | **2.601** |
| **CHRNB4** | 0.50 | **2.395** | **2.343** | **1.553** | **3.028** |
| **CHRNA3** | 1.00 | 0.774 | 1.356 |  | 0.882 |
| **CHRNA5** | 1.00 | **1.720** | **4.755** | **1.505** | **4.780** |
| **CHRNA6** | 1.00 | **9.451** | **9.533** | **2.794** | **9.731** |
| **CHRNA7** | 1.00 | 0.752 | 1.261 | 0.598 | 0.790 |
| **CHRNB2** | 1.00 | 1.814 | 1.621 |  | **1.621** |
| **CHRNB4** | 1.00 | 0.406 | 1.523 | 0.784 | **6.564** |

Values are mean fold change in mRNA expression in HBO cells (Fig 13) and STC-1 cells [5] relative to control (0 Nic); d = days
