# Supplementary figures and images for: Nicotinic acetylcholine receptor (CHRN) expression and function in cultured human adult fungiform (HBO) taste cells
Source: PLoS One. 2018 Mar 7;13(3):e0194089. doi: 10.1371/journal.pone.0194089 (PMC5841828; doi:10.1371/journal.pone.0194089)

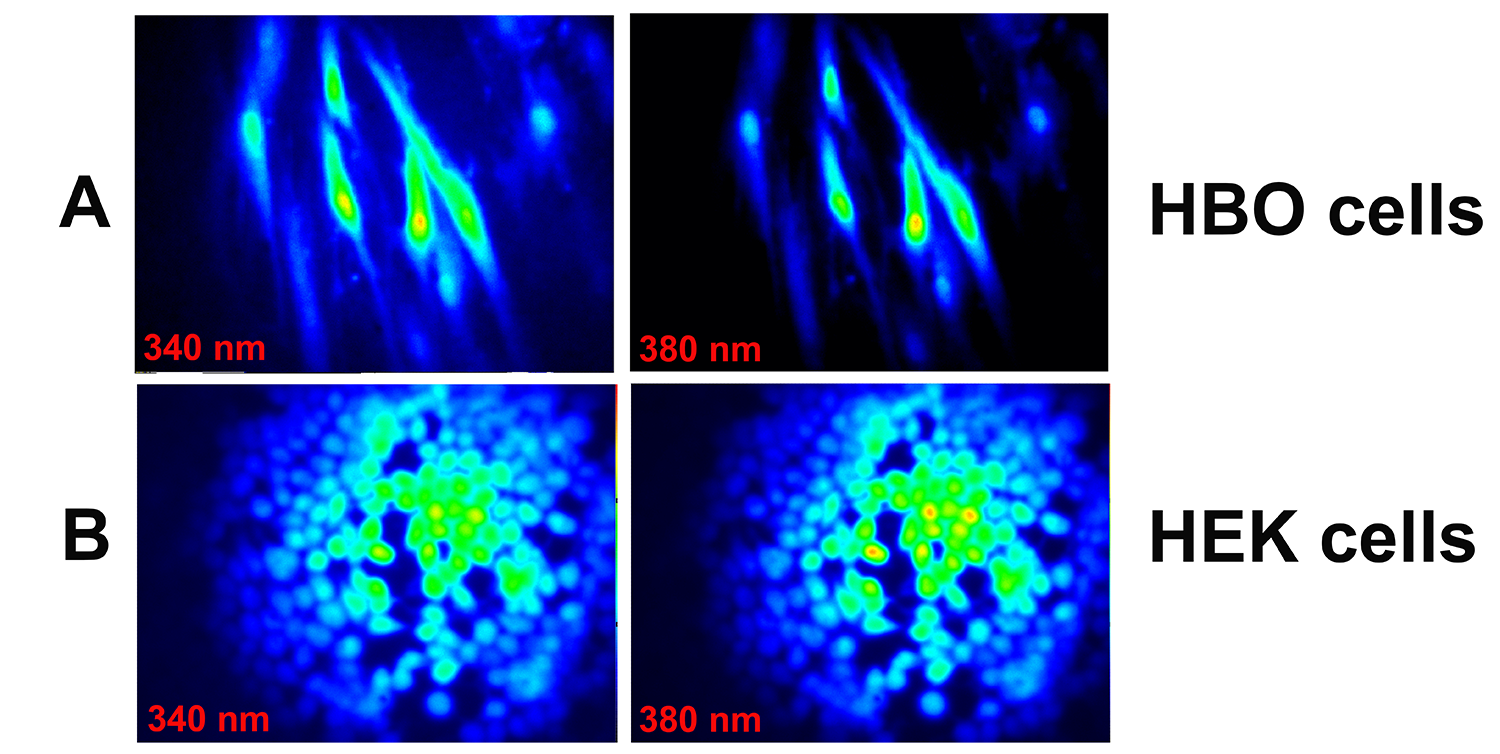

Supplement: S1 Fig — HBO and HEK293 cells were loaded with Fura-2. (A) Shows images of Fura-2 loaded HBO cells using excitation wavelengths of 340 and 380 nm. (B) Shows images of Fura-2 loaded HEK293 cells using excitation wavelengths of 340 and 380 nm. (TIF) [file pone.0194089.s003.tif]
